# Polymersome-based nanomedicine for combined cancer therapy

**DOI:** 10.1080/10717544.2025.2577137

**Published:** 2025-12-22

**Authors:** Aidin Mohammadi Zonouz, Mehrshad Ebrahimpour, Kimia Zibaei, Mohammad Ramezani, Sahar Taghavi, Khalil Abnous, Seyed Mohammad Taghdisi, Mona Alibolandi

**Affiliations:** a Pharmaceutical Research Center, Pharmaceutical Technology Institute, Mashhad University of Medical Sciences, Mashhad, Iran; b Student Research Committee, Mashhad University of Medical Sciences, Mashhad, Iran; c Department of Pharmaceutical Biotechnology, School of Pharmacy, Mashhad University of Medical Sciences, Mashhad, Iran; d Targeted Drug Delivery Research Center, Pharmaceutical Technology Institute, Mashhad University of Medical Sciences, Mashhad, Iran

**Keywords:** Nanomedicine, chemotherapy, dynamic therapy, photothermal therapy, immunotherapy, starvation therapy

## Abstract

Cancer is a complex disease characterized by uncontrolled cell growth and spread, often resulting in metastasis. The use of combination therapy, which involves the simultaneous application of two or more treatment methods, aims to enhance effectiveness and improve patient outcomes. This approach can enhance efficacy by targeting multiple mechanisms of cancer progression, leading to improved tumor response rates and survival. When combination therapy utilizes a codelivery platform, it enables dose reduction of each agent, thereby reducing side effects and increasing the therapeutic index. The simultaneous delivery of two or more drugs and therapeutic agents to solid tumors remains a major challenge in the development of more effective treatments. Polymersomes, owing to their unique and beneficial properties, are increasingly viewed as promising carriers for this purpose. They provide an excellent platform for combination therapies in cancer treatment. This review summarizes polymersome-based combination therapies, including chemotherapy, radiotherapy, antiangiogenic therapy, magnetic hyperthermia, dynamic therapy, starvation therapy, immunotherapy, photothermal therapy (PTT), and gene therapy. It also highlights recent advances and future prospects in polymersome-based combination cancer treatments. The aim of this study was to explore the challenges and opportunities in using polymersomes as drug codelivery vehicles, thereby enhancing our understanding of nanomedicine, especially in combination cancer therapies. Polymersome-based combination therapies have the potential to significantly transform current cancer treatments by enhancing accessibility, precision, and effectiveness while minimizing off-target effects.

## 1. Introduction

The World Health Organization (WHO) defines cancer as a wide range of diseases described by the rapid growth of abnormal cells. These cells can be found in nearly every organ or tissue of the body and are associated with various types of cancer. The defining feature of cancer cells is not only that they grow uncontrolled but also that they penetrate through the usual boundaries into neighboring tissues and can migrate to other parts of the body through a process called metastasis. The invasion of adjacent tissues by cancer cells differentiates them from benign tumors, which do not invade surrounding tissues. Understanding this definition is crucial for developing effective oncology prevention, diagnostic, and therapeutic strategies (Health topics: cancer).

Recent advances in cancer research and treatment include progress in immunotherapy, targeted therapies, precision oncology, early detection techniques, and personalized medicine. These breakthroughs are transforming the way cancer is detected, diagnosed, and treated, resulting in more effective and personalized approaches. Innovations such as synthetic biology, CRISPR, and the rise of data from AI and machine learning are seen as game changers. They offer unprecedented opportunities to better understand and combat cancer (Khan et al. 2024).

**Figure 1.**
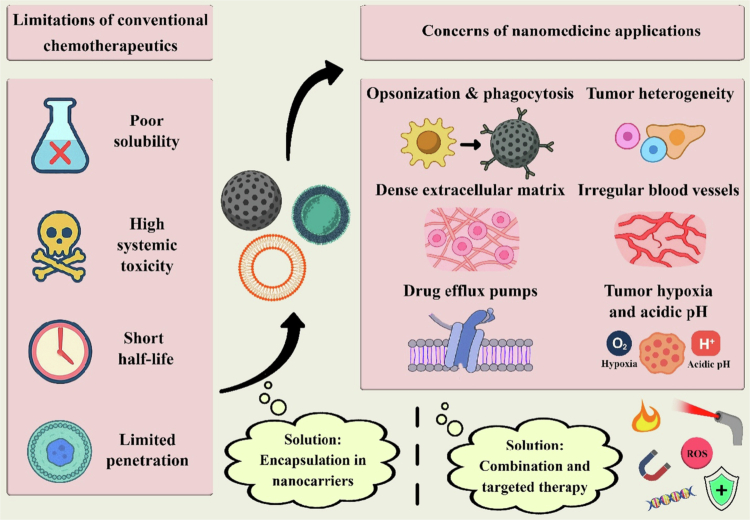
Challenges and solutions in cancer chemotherapy and nanomedicine applications. Conventional chemotherapeutics face several limitations, including poor solubility, high systemic toxicity, short half-life, and limited ability to penetrate tumors. Encapsulating drugs in nanocarriers can increase their therapeutic effectiveness. However, nanomedicine also encounters obstacles such as opsonization and phagocytosis, tumor heterogeneity, a dense extracellular matrix, abnormal blood vessel formation, drug efflux pumps, and the hypoxic and acidic microenvironment of tumors. Strategies to address these issues include combination and targeted therapies, along with optimized nanocarrier design, to improve drug delivery and treatment outcomes. The original image was drawn via Adobe Photoshop.

Cancer can be fatal if left untreated. However, many severe complications are associated with cancer treatment that may be life-threatening if not addressed quickly and effectively. The most significant complications include cardiovascular issues (Bohdan et al. 2021; Narayan and Ky 2018), oral problems such as mucositis, xerostomia, and dysphagia (Abo el Fadel et al. 2024; Naidu et al. 2004), gastrointestinal side effects such as diarrhea, constipation, nausea, vomiting, and abdominal pain (Akbarali et al 2022), myelosuppression (Hart et al. 2023; Joudeh et al. 2023), and neurological complications (Alberti et al. 2022; Jones et al. 2020). There are many obstacles to the effective use of chemical agents, including poor solubility, limited penetration, high systemic toxicity, and a short half-life in the blood circulation. In this context, encapsulating anticancer agents in nanoparticles could improve the therapeutic index (Hafeez et al. 2021; Paresishvili and Kakabadze 2023). However, nanoparticles also present numerous challenges that must be addressed through careful design and engineering. For example, nanoparticles (NPs) are cleared from the circulation via opsonization and phagocytosis by mononuclear phagocytic cells (Li et al. 2023; Sriraman et al. 2014). Additionally, the abnormal structure of tumor blood vessels creates significant difficulties; these irregular vessels cause unpredictable drug distribution and can hinder delivery to specific tumor areas (Azzi et al. 2013). The dense extracellular matrix surrounding tumor cells further reduces the drug delivery space, limiting drug penetration into tumors (Huang et al. 2021; Subrahmanyam and Ghandehari 2021). Moreover, drug efflux pumps, such as *P*-glycoproteins (*P*-gp), actively expel drugs from cells, while hydrophilic drugs and larger molecules face barriers crossing cell membranes (Ughachukwu and Unekwe 2012; Xue and Liang 2012). The tumor microenvironment, characterized by hypoxia and acidic pH, can significantly influence drug effectiveness and activity (Sriraman et al. 2014; Ghaemi and Javad Hajipour 2023; Hompland et al. 2021). Other major barriers include tumor heterogeneity, where different regions within the tumor respond variably to treatment and often differ from metastatic sites that stem from the primary tumor (Dagogo-Jack and Shaw 2018). Given these numerous obstacles, various strategies are under exploration (Figure 1), including nanocarrier systems, targeted therapies, and combination approaches (Li et al. 2023; Lorscheider et al. 2021; Yao et al. 2020).

Medical nanotechnology addresses challenges in drug delivery. This approach is believed to improve key features of drug delivery methods by optimizing controlled and sustained release, increasing encapsulation efficiency, improving biological system stability, and increasing the bioavailability of therapeutic agents (Mohammadi et al. 2017). Nanoparticles (NPs), defined as particles with at least one dimension between 1 and 100 nm, have unique properties that differ from those of bulk materials of the same composition and larger sizes (Gavas et al. 2021). Their high ability to accumulate in tumors enhances the enhanced permeation and retention (EPR) effect. Furthermore, the surface properties of these NPs significantly improve their potential for bioavailability and half-life, enabling them to pass through epithelial fenestrations (Shin et al. 2016). The organic NPs used in drug delivery include polymeric NPs, dendrimers, monoclonal antibodies, extracellular vesicles, liposomes, solid lipid NPs, nanoemulsions, and cyclodextrin nanosponges. In contrast, inorganic NPs include carbon-based NPs, quantum dots, magnetic NPs, calcium phosphate NPs, and silica NPs. The field also benefits significantly from hybrid NPs (Gavas et al. 2021; Krishna et al. 2024; Lavania et al. 2023; Mehta et al. 2024; Ponnamma et al. 2016).

**Figure 2.**
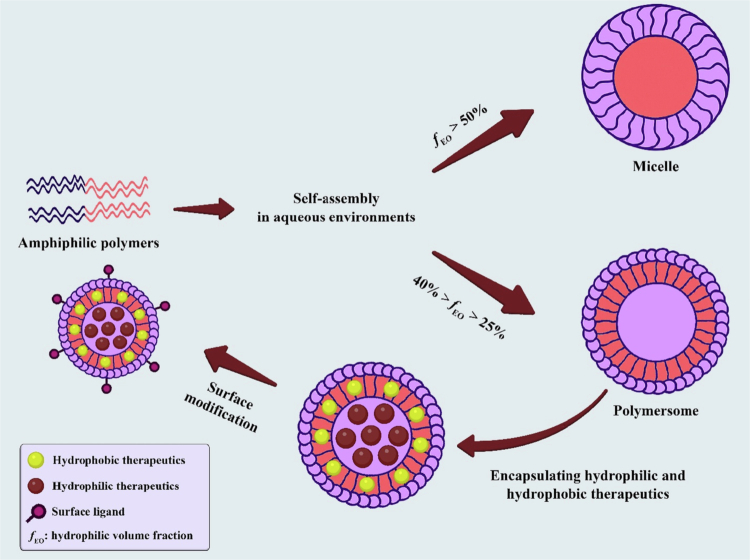
Self-assembly of amphiphilic polymers into micelles and polymersomes in aqueous environments. Micelles form when the hydrophilic volume fraction (*fEO*) exceeds 50%, while polymersomes form at 25% <*fEO* <40%. Polymersomes can carry both hydrophilic and hydrophobic drugs and can be modified on the surface with ligands for targeted drug delivery. The original image was drawn via Adobe Photoshop.

Self-assembly is observed in various types of nanocarriers, including spherical and worm-like micelles, as well as polymersomes, formed through self-assembly in water-driven amphiphilic copolymers (Gref et al. 1994). One striking property of all amphiphilic polymers is related to the critical aggregation concentration, also known as the critical micellization concentration. The concentration of amphiphilic polymers in aqueous media above which they begin to self-assemble into different aggregate structures (such as micelles and polymersomes) has been termed the critical aggregation concentration (CAC) or critical micelle concentration (CMC) (Yetisgin et al. 2024). Polymersomes are self-assembled polymeric vesicles that can deliver drugs in a biologically stable manner (Gavas et al. 2021). This is because of their morphological similarity to those of cellular membranes and viral capsids (Araste et al. 2021). The properties of a drug, including its encapsulation and release, can be readily adjusted by utilizing a variety of biodegradable and/or stimulus-responsive block copolymers (Han et al. 2023). Compared with that of liposomes, the enhanced stability of polymersomes presents opportunities for achieving more precise release kinetics. Further advances in nanomedical formulations would include triggering release only at the site of action. Polymersomes are spherical nanostructures characterized by their hollow interiors and bilayer membranes, which consist of hydrophobic segments paired with hydrophilic crowns on either side. These structures can encapsulate water-soluble substances within their central cavity while also hosting hydrophobic molecules within their bilayer membranes. Owing to the higher molecular weights of the materials that make up their structure and the increased thickness of their vesicular membranes, polymersomes exhibit greater chemical and mechanical stability than liposomes do. These characteristics of polymersomes make them highly suitable platforms for codelivering therapeutic molecules in combination with cancer therapy. For instance, polymersomes made from poly(butadiene-ethylene oxide) (PB-PEO), when loaded with paclitaxel (PTX), demonstrated a consistent release profile over five weeks at 37 °C, which was associated with a decrease in cytotoxic effects (Zhang and Zhang 2017). Polymersomes can encapsulate hydrophilic, hydrophobic, or amphiphilic compounds, making them highly attractive vesicles for various applications, including drug delivery, such as transporting anticancer compounds, and biomedical imaging and diagnostic processes (Massignani et al. 2010). Polymersomes possess a membrane thickness several times greater than that of liposomes because block copolymers have higher molecular weights than phospholipids do (Chandrawati and Caruso 2012). This feature provides polymersomes with improved colloidal stability, enabling them to better resist osmotic pressure and mechanical shear forces during blood circulation (Mohammadi et al. 2017). The chemistry of amphiphilic copolymers offers easier modification strategies for surface modification of polymersomes with specific ligands, enabling the selective delivery of anticancer medications by interacting with receptors overexpressed on cancerous cells (Thambi and Lee 2019). Polymersomes and micelles are both spherical self-assemblies of amphiphilic copolymers in aqueous environments and are widely used in drug delivery applications. It has been demonstrated that micelles and polymersomes are formed from linear amphiphilic copolymers (Figure 2) with hydrophilic volume fractions (*f*_EO_) greater than 50% and 25% < *f*_EO_ < 40%, respectively (Mohammadi et al. 2017). A previous study demonstrated that the loading capacity and encapsulation efficiency of polymersomes were greater than those of micelles. This could be due to the greater molecular weight of the hydrophobic section in the polymersome structures and the larger size of the polymersomes than the micelles. Additionally, the shorter hydrophobic blocks in micelles allowed cargo to be released more quickly than those in polymersomes. Owing to this characteristic, water can more easily diffuse into the NP, leading to micelle destruction and structural instability (Alibolandi et al. 2015).

We are currently witnessing a series of groundbreaking technological advances in cancer research. These developments have enhanced our understanding of tumors and their microenvironments, revealing numerous new therapeutic targets. In advanced cancers, therapy resistance often reduces the effectiveness of single-agent treatments. As a result, approximately 5,000 clinical trials are ongoing worldwide to explore the benefits of new combination therapies. However, the possible combinations of treatments far outnumber the patients available for these trials. This gap may lead to drawbacks such as missed opportunities, trial failures, unnecessary toxicity, poor patient enrollment, and financial losses. One way to address this issue could be through more strategic design of combination therapies, based on key biological and mechanistic insights.

Combination therapy in cancer treatment involves the simultaneous use of two or more treatment methods to increase effectiveness and improve patient outcomes (Liu et al. 2019). It is one of the most commonly used approaches in clinical settings for cancer therapy. This strategy typically involves the administration of multiple anticancer drugs that work through distinct mechanisms. This can help slow the ability of tumors to adapt and reduce the development of mutations in cancer cells. Additionally, it enhances effectiveness through synergistic effects and increases target selectivity when multiple drugs are used against the same cellular pathway (Xu et al. 2017).

Combination therapy utilizing drug delivery platforms involves two primary approaches: (1) the application of one drug-loaded delivery platform in combination with another drug-loaded delivery platform and (2) a codelivery platform that contains multiple therapeutic agents within a single delivery platform (Li et al. 2015).

Combination cancer therapies offer several benefits. They can increase treatment efficacy by targeting different mechanisms of cancer progression, improving tumor response rates and survival. Some combinations provide the advantage of reducing the dose of each agent, thereby minimizing adverse side effects; however, a few can actually increase toxicity. The second benefit is synergism: in this context, many combinations achieve a better overall outcome because their combined effect is much greater than the sum of what each would produce in separate trials. Additionally, the use of multiple agents may prevent or delay resistance, a problem generally associated with single-agent therapies. Finally, combination therapies are advantageous because they enable more personalized treatments by considering specific patient characteristics, such as the tumor profile and genetic markers, to ensure a tailored approach to care (Mokhtari et al. 2017; Sicklick et al. 2019).

In recent years, the administration of polymersomes has emerged as a potential strategy in combination cancer therapy. This is achieved by overcoming multidrug resistance (Han et al. 2023; Li et al. 2015), producing a synergistic therapeutic effect (Noh et al. 2015; Wu et al. 2023a; Zhu et al. 2015), reducing the required dosage (D'Angelo et al. 2022), and mitigating the adverse effects of chemotherapeutic agents (Anajafi et al. 2017). Current cancer therapies are usually classified into traditional and modern approaches. The three most prevalent traditional cancer therapy techniques (chemotherapy, radiation therapy, and surgery) have several disadvantages (Beik et al. 2016). Most contemporary methods, including dynamic therapy, immunotherapy, magnetic hyperthermia, photothermal therapy, gene therapy, and starvation therapy, are employed to treat cancer. Polymersome-based combination therapies for cancer, which include chemotherapy, radiotherapy, antiangiogenic therapy, magnetic hyperthermia therapy, dynamic therapy, starvation therapy, immunotherapy, photothermal therapy (PTT), and gene therapy, are summarized in this review.

## 2. Method

In this review article, a comprehensive search was conducted across several databases, including PubMed, Google Scholar, and Web of Science, without time limitations. The search yielded a wide variety of articles, including *in vitro* and *in vivo* research. Several search terms were used to ensure a comprehensive exploration of the subject, including ‘polymersome,’ ‘drug delivery systems,’ ‘nanomedicine,’ ‘therapeutic cargo,’ ‘targeted therapy,’ ‘polymersome formation method,’ ‘combination therapy,’ ‘dual therapy,’ ‘chemotherapy,’ ‘dynamic therapy,’ ‘photothermal therapy,’ ‘immunotherapy,’ and ‘gene therapy.’

## 3. Polymersome-based combination therapy

### 3.1. Combination of chemotherapeutics

Combination chemotherapy, the simultaneous use of two or more chemotherapeutic drugs has been shown to produce effective therapeutic results in mice with advanced leukemia since the 1960s. Later, similar results were subsequently reported in patients with advanced diffuse histiocytic lymphoma. Many combination regimens have been developed over the past few decades to treat multiple types of tumors. The following general concepts can generally serve as the foundation for therapeutic combinations: 1) without overlapping toxicities, which allows each drug to be given at an appropriate dose; 2) with distinct therapeutic mechanisms and low levels of cross-resistance, which suppresses a wide range of drug resistance; 3) with dose ratios optimized for synergistic/additive therapeutic effects; and 4) with comparable solubility and permeation to guarantee adequate delivery and high intracellular concentrations (Qin et al. 2018).

Combination chemotherapy is the top clinical choice for treating cancer. In this context, researchers have designed drug combinations and conducted studies using in vitro optimization to increase drug effectiveness and enhance synergistic effects. However, translating these combinations into successful in vivo treatments is challenging because of differences in how the drugs are absorbed and metabolized. By encapsulating each drug pair in polymersomes, we can achieve consistent pharmacokinetics across the combinations, making it possible to evaluate the inherent benefits of these drug combinations.

In this context, researchers have developed various polymersomes based on polyethylene glycol-polyesters for the coencapsulation of anticancer agents.

D'Angelo et al. fabricated polyethylene glycol-poly(*ε*-caprolactone) (PEG-PCL)-based polymersomes comprising PEG_45_PCL_44_, PEG_114_PCL_98_, and PEG_114_PCL_114_ block copolymers in a study. Two distinct chemotherapeutic agents were coencapsulated in PEG-PCL-based polymersomes: doxorubicin (DOX), a hydrophilic drug used in the treatment of hematological cancers and solid tumors, and vemurafenib (VEM), a hydrophobic drug for the treatment of metastatic malignant melanoma. The polymersomes exhibited no instability at 4 °C, 25 °C, and 37 °C. Furthermore, formulations containing smaller PEG-PCL blocks (PEG_45_PCL_44_ > PEG_114_PCL_98_ > PEG_114_PCL_114_) demonstrated faster drug release. In contrast to the physiological pH of 7.4 at 37 °C, these PEG-PCL-based polymersomes exhibited improved drug release under mildly acidic conditions (pH 5.0 at 37 °C, mimicking the tumor microenvironment). This study presents an innovative codelivery approach that employs different antitumor mechanisms, aiming to increase the therapeutic index. This strategy could enable lower dosages and mitigate the risk of multidrug resistance (MDR) (D'Angelo et al. 2022).

Verapamil hydrochloride was used in another investigation to suppress the expression of *P*-gp. In an attempt to increase the anticancer impact of DOX and reverse MDR by blocking *P*-gp expression, hydrophilic DOX and verapamil were loaded into mPEG-PCL-poly(glutamic acid) (PGA) polymersomes. *In vitro* cytotoxicity investigations demonstrated that the produced polymersomes exhibited a greater inhibition ratio against adriamycin-resistant MCF-7 cells than both polyDOX and the free DOX solution. Compared to the free drug solutions, the release rates of the two medicines from poly(DOX + verapamil) were significantly lower, and their release profiles showed strong pH sensitivity. Additionally, the low hemolysis rate of mPEG-PCL-PGA demonstrated that the copolymer could be used safely for intravenous injection (Li et al. 2015).

While polymersomes can encapsulate larger amounts of both hydrophobic and hydrophilic drugs, controlling the gradual release of these substances remains challenging. In this context, Nahire et al. developed and characterized folate-targeted air bubbles, gemcitabine, and DOX-loaded echogenic PEG-s-s-PLA polymersomes designed for cytosolic delivery to cancer cells, primarily targeting pancreatic and breast cancer cells. This platform may offer greater stability compared to traditional drug delivery systems, such as liposomes. To address this issue, this research engineered an echogenic polymersome that responds to cytosolic GSH levels, enabling the rapid release of encapsulated gemcitabine and DOX owing to the properties of their block copolymer. Additionally, the fabricated polymersomes encapsulated air bubbles, enhancing their echogenic capabilities for ultrasound imaging and ultrasound-triggered release. The study demonstrated that folate-targeted polymersomes significantly improved drug uptake in both monolayer and three-dimensional spheroid cultures of cancer cells (PANC-1 and MCF-7). This research suggests that polymersomes can quickly release their contents in response to reducing agents, mimicking the intracellular environment. Ultrasound-induced release studies revealed that while ultrasound alone did not substantially increase drug release, combining ultrasound with reducing agents produced a synergistic effect. Overall, these findings suggest that the use of multifunctional polymersomes could be a promising approach to enhance the delivery and efficacy of gemcitabine and DOX in cancer therapy. Furthermore, they hold potential for development into clinically relevant drug delivery systems capable of diagnostic imaging (Nahire et al. 2014).

In another investigation, gemcitabine and DOX were delivered to the nucleus of pancreatic cancer cells concurrently using a nuclear-targeted, redox-sensitive drug delivery system. In this context, PEG-s-polylactic acid (PLA), a redox-sensitive copolymer, was used to formulate a polymersomal coloaded platform to increase the sensitivity toward a glutathione-rich environment. Acridine orange, which intercalates DNA and has inherent affinity for it, was conjugated to the polymer surface to impart nuclear localization capability. By guided delivery of the co-loaded drugs to the nucleus, the fabricated platform illustrated a high therapeutic index *in vitro* toward spheroids of pancreatic cancer (Anajafi et al. 2016).

Tariquidar (TQR) is an inhibitor of *P*-gp used in combination cancer therapy. TQR binds to *P*-gp and inhibits its ability to pump anticancer agents out of cells. In addition, its combination with other anticancer agents could increase the therapeutic index. In this context, TQR, DOX, and PTX have been incorporated into a folate-decorated PCL-ss-PEG-ss-PCL-based redox-responsive polymersome. Following folate receptor-mediated endocytosis, glutathione (GSH) in the cytosol cleaves disulfide bonds in formulated polymersomes, triggering the rapid release of TQR, DOX, and PTX. Drug concentration in adriamycin-resistant MCF-7 cells by TQR-induced *P*-gp efflux inhibition was increased when folate-targeted polymersomes were used *in vitro*. This approach is expected to improve and strengthen the cytotoxic effects and proapoptotic activity against drug-resistant tumor cells (Qin et al. 2018).

Thambi et al. synthesized a PEG-b-polylysine (PLys)-s-PCL-based bioreducible, amphiphilic triblock copolymer. The amphiphilicity of the copolymer facilitated its self-assembly into polymersomes in an aqueous environment. The polymersomes simultaneously encapsulated the hydrophilic DOX and the hydrophobic camptothecin (CPT), releasing the cargos in a redox-responsive manner. The results revealed noticeably greater cytotoxicity of the platform against SCC7 cancer cells compared to the single-drug-loaded system (Thambi et al. 2012). In another attempt to overcome drug resistance by blocking *P*-gp expression and enhancing the accumulation of encapsulated anticancer agents, a combination drug delivery platform incorporating a reduction-sensitive PTX polymeric prodrug (PEG-b-poly(2-methacryloyloxyethyl phosphorylcholine) (PMPMC)-g-PTX) (PMP) polymersomes was developed. This PTX polymeric prodrug self-assembled into a polymersome and was coloaded with DOX·HCl and the *P*-gp inhibitor Tariquidar (TQR). PTX detaches from the PMPMC chain when disulfide bonds break in a reductive glutathione-rich environment (the cytoplasm of cancer cells and the tumor microenvironment). Then, the balance between the hydrophilic and hydrophobic blocks is disrupted; the cargos, DOX·HCl and TQR, are released more quickly because the polymersomes rupture. The results revealed that the prepared combination drug codelivery system, PMP/DOX·HCl/TQR, had a strong growth-inhibitory effect on adriamycin-resistant MCF-7 cells or nonresistant HeLa cells (Xu et al. 2017).

Rapamycin, also known as sirolimus, is an anticancer and immunosuppressive drug that inhibits mTOR protein kinase, a key component of a signaling cascade involved in cell survival and growth. The pathway is often disrupted in cancer cells, making mTOR an attractive target for cancer treatment (Bouyahya et al. 2022). OXA is an anticancer drug that interferes with DNA replication and transcription via the formation of nuclear DNA adducts. OXA-DNA complexes can trigger apoptotic or DNA repair pathways (Sepay et al. 2024). OXA has also been shown to exert a synergistic effect when combined with RAPA by inhibiting the mammalian target of rapamycin (mTOR) pathway (Li et al. 2016). However, RAPA is less water soluble, is very unstable in gastric juice, and is usually administered intravenously. Intravenous administration is often painful and requires the assistance of medical personnel for proper administration. In contrast, oral administration is convenient and enhances compliance with treatment (Yang et al. 2024).

**Figure 3.**
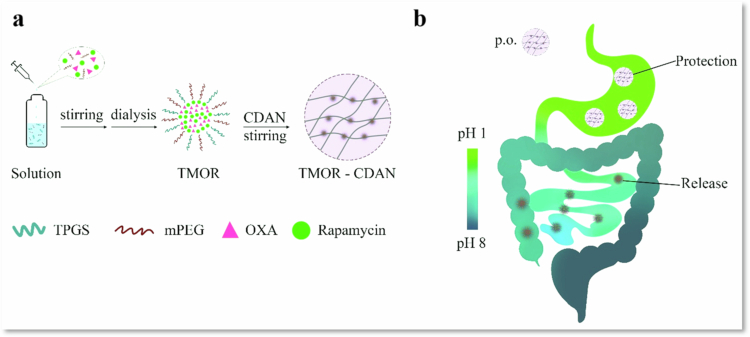
(a) Development of TMOR-CDAN. (b) Protecting drugs from harsh gastrointestinal conditions and ensuring their release in the lower intestines. The figure was redrawn by the authors using Adobe Photoshop, which is based on information from Wande et al. (2022).

In this regard, a novel drug delivery system, a composite of nanogel-polymersome called TMOR-CDAN, was created for the simultaneous oral delivery of oxaliplatin (OXA) and rapamycin, aiming for synergistic chemotherapy. This novel approach overcomes the two main hurdles in cancer treatment, i.e., the drug resistance problem and the low solubility of rapamycin, which have limited its clinical use. The TMOR-CDAN system utilizes chitosan diacetate (CDA) derivatized with mPEG and TPGS to create a strong and effective formulation capable of encapsulating both hydrophilic OXA and hydrophobic rapamycin. The unique features of this system enable it to remain stable across a wide range of pH values, from acidic to alkaline, making it highly suitable for use in harsh gastrointestinal environments. The TMOR-CDAN formulation results in a controlled and prolonged drug release profile, a crucial parameter for maintaining therapeutic concentrations of the drug throughout the gastrointestinal tract. This is particularly advantageous for rapamycin, which is susceptible to instability at acidic pH. *In vitro* studies revealed that TMOR-CDAN significantly increased cytotoxicity in different cancer cell lines, including 4T1 and B16F10. In *in vivo* tests using a 4T1 tumor-bearing BALB/c mouse model, the TMOR-CDAN formulation showed higher antitumor efficacy than monotherapies or a combination of the two single agents. The synergistic effect of the OXA and rapamycin combination is exploited, where rapamycin enhances the sensitivity of cancer cells to OXA, resulting in increased apoptosis and decreased tumor proliferation. The findings of this study show that the TMOR-CDAN platform is dual-acting, protecting encapsulated chemotherapeutic agents from degradation in harsh gastric environments while enhancing their bioavailability. This innovative delivery platform represents a significant advancement in the field of combination chemotherapy regimens, as it offers an oral route of administration with the potential to substantially increase patient compliance and clinical treatment outcomes. This work highlights the potential of TMOR-CDAN as a multifunctional system for the effective codelivery of poorly water-soluble anticancer therapeutics, laying the groundwork for future research and applications in oncological therapy (Figure 3) (Wande et al. 2022).

In another study, a biodegradable methyl-PEG-PLA polymersome coated with a dual-functionalized tocopheryl polyethylene glycol succinate (TPGS)-lactobionic acid conjugate (TLA) was fabricated to reverse MDR through targeted administration of DOX and oxaliplatin. Tocopheryl polyethylene glycol succinate (TPGS)-lactobionic acid conjugate (TLA), which is attached to the surface of the polymersome, TPGS acts as a *P*-gp inhibitor (TPGS), and lactbionic acid acts as an ASGPR (asialoglycoprotein receptor) ligand, which endows the polymersome with the capacity to target hepatocellular carcinoma and accumulate inside tumor cells through the combined effects of *P*-gp inhibition and receptor-mediated endocytosis. In the preclinical stage, the EPR-mediated passive and TLA-driven active tumor-targeting effects result in the accumulation of DOX- and OXA-coloaded polymersomes at the tumor site following intravenous injection. In this context, the synergistic effect was maximized by codelivering DOX and OXA through a single nanovehicle, which reduces the pharmacokinetic profile variations between the two medications (Liu et al. 2019).

In another work, the systemic delivery of an anticancer cocktail using biodegradable PEG-PLA/PEG-butadiene (PBD) polymersomes was assessed *in vivo*. The prepared polymersomes effectively transport DOX and PTX, which act as hydrophilic and hydrophobic agents, respectively. In PLA/PEG-butadiene (PBD) polymersomes, the butadiene (PBD) block is responsible for forming the hydrophobic core of the vesicle bilayer membrane. This hydrophobic segment could provide a high capacity for the encapsulation and delivery of hydrophobic molecules, while the hydrophilic PEG block provides biocompatibility and stability. The properties of the PBD block, such as its length, can be adjusted to control the thickness of the membrane and its loading capacity for hydrophobic drugs. In addition to long-term circulation *in vivo*, polymersomes can release their cargos within a day. A single systemic injection of the prepared formulation in MDA-MB231 tumor-bearing nude mice resulted in a high maximum tolerated dose. Additionally, it achieves a more effective therapeutic index, with a 50% reduction in tumor size within 5 days when treated with polymersomes. Fabricated polymersomes have the potential for use in a multidrug delivery platform with a quantitatively comparable increase in the maximum tolerated dose and drug accumulation in tumors (Ahmed et al. 2006).

We et al. reported a folate (FA)-conjugated Pluronic F127/poly(D,L-lactide-b-glycolide) (FA-F127-PDLLAGA) polymersome to facilitate the simultaneous delivery of PTX and DOX, aiming to increase their anticancer efficacy. Polymersomes release PTX and DOX in a controlled manner. The *in vitro* cytotoxicity results revealed that compared with single-loaded polymersomes and free drugs, targeted co-loaded polymersomes had greater inhibitory effects. Furthermore, formulated polymersomes have synergistic therapeutic effects while the folate-modified polymersomes have superior targeting capabilities (Wu et al. 2023a, 2023b).

A therapeutic polymer that is composed of polymer‒drug conjugates. In some cases, these therapeutic polymers exhibit amphiphilic properties and can self-assemble into various structures, including polymersomes, micelles, and other complex forms. In this context, PTX and platinum (Pt) were conjugated to the PGA-PEG-PGA triblock copolymer. Notably, PGA-PEG-PGA is soluble entirely in water, but after Pt and PTX conjugation, it forms a polymersome via self-assembly. After drug release, the hydrophobicity of the platform decreased, and the PGA-PEG-PGA copolymers switched to water-soluble copolymers. The synergistic effects of Pt and PTX on a lung cancer cell line (A549) and a prostate cancer cell line (LNCaP) are shown. The impact of dual drug delivery in 3D multicellular spheroids was also examined. The synergistic impact of the combination of Pt and PTX in the prepared platform was validated by Chou-Talalay analysis (Callari et al. 2017).

**Figure 4.**
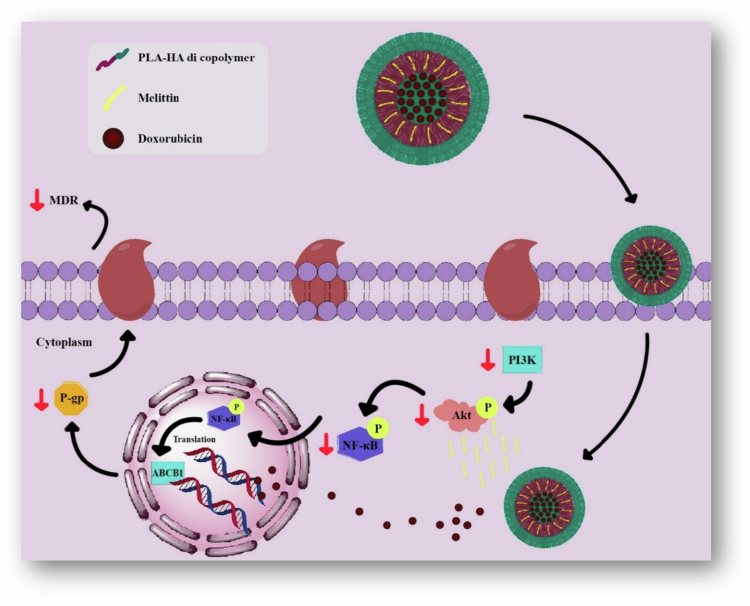
Codelivery of melittin and DOX through a PLA-HA polymersome targets the downregulation of PI3K/Akt and NF-κB pathways, thereby overcoming MDR and improving chemotherapeutic effectiveness. The figure was redrawn by the authors using Adobe Photoshop, based on information from Han et al. 2023).

Overexpression of efflux receptors such as *P*-gp is a major obstacle to chemotherapy because they pump out intracellularly delivered antitumor agents. The main component of bee venom, Mel, can inhibit *P*-gp-mediated MDR-related pathways, including the PI3K/Akt and NF-κB pathways, in cancer cells such as adriamycin-resistant MCF-7 cells. The significance of the PI3K/Akt pathway lies in its ability to inhibit cell apoptosis and increase cancer cell proliferation, invasion, and angiogenesis. For this reason, the delivery of DOX-Mel-loaded polymersomes represents a novel approach to improve the efficacy of chemotherapeutic agents against MDR cells. In adriamycin-resistant MCF-7 and other MDR cells, there is a high expression of the CD44 receptor. Targeting MDR cells is made possible by HA, which is the most specific ligand for CD44 activation. Given these findings, Melittin (Mel) and DOX were coencapsulated into the PLA-hyaluronic acid (HA) polymersome. Formulated polymersomes improve the efficacy of chemotherapy and overcome MDR through the coencapsulation of drugs. The suppressant efflux pump by Mel results in high intracellular DOX concentrations, which subsequently stimulate cell apoptosis (Figure 4) (Han et al. 2023).

A versatile approach was also developed to target non-small-cell lung cancer by constructing a self-targeted polymersomal coformulation based on an amphiphilic HA-b-PCL diblock copolymer. This innovative formulation was designed to encapsulate DOX and CPT in distinct compartments of the polymersome, enhancing therapeutic efficacy and reducing systemic toxicity. The characterized HA-b-PCL copolymer demonstrated the ability to form polymersomal structures that effectively coencapsulated DOX and CPT at 1:10 and 1:1 ratios. Moreover, the decoration of the hyaluronic acid coating on the polymersome surface achieved an active targeting effect toward CD44 receptors, which are reported to be overexpressed on the membranes of cancer cells, thereby enhancing both the specificity and efficiency of the drug delivery system. Moreover, the conjugation of the FOXM1-specific DNA aptamer (which inhibits FOXM1 transcriptional activity) to the polymersome surface enhanced the cytotoxic effect, thereby promoting the apoptosis of cancer cells. Notably, the use of formulated polymersomes demonstrated a significant tumor-suppressing effect in SK-MES-1 tumor-bearing C57BL/6 nude mice. Additionally, the sustained and controlled release profiles of the encapsulated drugs, extending for up to 200 hours, demonstrated the potential of the HA-b-PCL nanocarrier to provide long-term therapeutic effects (Shahriari et al. 2021).

A stimuli-responsive delivery system could provide on-demand drug release and provide remarkable therapeutic efficacy. In this context, a photocrosslinked polymersome that responds to both temperature and pH was designed through the self-assembly of a triblock polymer of mPEG-b-poly(*N*-isopropylacrylamide)-b-poly[2-(diethylamino)ethyl-methacrylateco-2hydroxy-4(methacryloyloxy)benzophenone] (mPEG-b-PNIPAM-b-P(DEAEMA-co-BMA)). PNIPAM served as the temperature-responsive component, PDEAEMA served as the pH-responsive segment, and BMA served as the photocrosslinker to enhance membrane stability. mPEG was added to improve the biocompatibility and prolong the circulation time in the bloodstream. Both hydrophilic DOX and hydrophobic PTX were coloaded into the resulting polymersomes, thereby eliminating monotherapy issues such as drug resistance and ineffective treatment of complex diseases and enhancing therapeutic efficacy. The polymersomes displayed favorable dual responsiveness to temperature and pH, with tunable temperature sensitivity at varying pH values, and thus are promising combination chemotherapy agents (Zhou et al. 2021).

In an investigation, three constituents, two hydrophobic molecules, such as a decyl chain and cholesterol, and a single hydrophilic molecule, PEG, self-assembled into a new electroneutral polymersome. The polymersomes generated were used as carriers for three anticancer drugs: DOX, 5-fluorouracil (5-FU), and leucovorin. The efficiency of loaded polymersomes was compared *in vitro* and *in vivo* with that of ectopic pancreatic BxPC-3 tumors in NOD SCID mice through both intratumoral and intravenous administration. Intratumoral administration of drug-loaded polymersomes resulted in a 60% reduction in tumor volume after 13 days. Intravenously, the polymersomes caused a 40% increase in tumor volume, which was better than the 53% increase observed with free drug solutions at day 7. Gene expression analysis of treated tumors revealed that the treatment induced apoptosis and decreased the expression of genes associated with tumor survival, suggesting a reduction in stem cells, angiogenesis, and hypoxia-induced stress (Aibani et al. 2018).

In another study, hydrophobic and hydrophilic chemotherapeutics, comprising PTX and DOX, were coencapsulated within a polymersome composed of a PCL_8000_-PEG_8000_-PCL_8000_ triblock copolymer. This nanosized platform was also tagged with folate to enhance targeted tumor therapy. *An in vivo* antitumor test confirmed that the intravenous delivery of polymersomes in combination with chemotherapy was superior to free-drug combination administration against BEL-7404 tumors and that the tumor growth inhibition of folate-targeted polymersomes was greater than that of nontargeted polymersomes. Folate-targeted polymersomes have promising applications in the codelivery of multidrug chemotherapy, with potential uses in tumor targeting and combination chemotherapy (Zhu et al. 2017).

Histone deacetylases are responsible for regulating gene expression and chromatin remodeling. Unusual expression or mutation of histone deacetylases can potentially cause cancer formation. Therefore, histone deacetylase targeting has proven to be an encouraging approach for cancer treatment. Histone deacetylase inhibitors, such as the class I/IV inhibitor mocetinostat, have shown encouraging antitumor effects, including anti-drug resistance and the inhibition of tumor growth and metastasis in various cancers (Lian et al. 2023). Karandish et al. synthesized a PEG-ss-PLA diblock copolymer to create reduction-sensitive polymersomes with folate on their surface, coencapsulating docetaxel and mocetinostat. Folate on the polymersome surface effectively targeted the prostate-specific membrane antigen (PSMA) receptor of prostate cancer cells. LNCaP cell (PSMA-positive) survival was significantly reduced in three-dimensional spheroid cultures by two drug-encapsulated polymersome formulations in contrast to free drugs. The calculated combination index values suggested a synergistic interaction between mocetinostat and docetaxel. PSMA-targeted drug-loaded polymersomes containing a histone deacetylase inhibitor (mocetinostat) and an anticancer drug (docetaxel) have been shown to be promising options for enhancing prostate cancer therapy by reducing systemic toxicity and maximizing the efficiency of drug delivery (Karandish et al. 2016).

Nisin is an antibacterial peptide produced by fermentation of the gram-positive bacteria Lactococcus lactis. The FDA and WHO have approved its use as a food preservative (Kamarajan et al. 2015). Recently, researchers have studied nisin as a potential anticancer agent. Reports indicate that nisin can induce apoptosis, leading to cancer cell death (Ahmadi et al. 2017). However, the clinical applications of nisin have been limited due to its low serum bioavailability, poor absorption, and limited biodistribution within the body (Biswaro et al. 2018; Khazaei Monfared et al. 2023). Its poor bioavailability is due primarily to its instability at physiological pH, susceptibility to proteolytic degradation, and ability to bind to serum proteins such as albumin, all of which limit its ability to reach target cells and function effectively as an antimicrobial.

Curcumin (CUR), a naturally occurring polyphenol that is separated from *Curcuma longa*, has anticancer and other pharmacological effects. The anticancer effects of CUR are attributed to the induction of apoptotic pathways in cancer cells, the suppression of tumor metastasis, and the modulation of inflammation and angiogenesis within the tumor microenvironment (Shakeri et al. 2019). Despite several therapeutic advantages, the physicochemical properties of CUR, including poor solubility, low oral bioavailability, poor adsorption, and rapid metabolism and elimination from the body, limit its potential medical applications (Hussain et al. 2017). A study employed a mixture of polyvinyl alcohol (PVA) and PEG in a 1:3 ratio [PVA-PEG graft copolymer (Kollicoat®)] and PCL to develop nisin and CUR-loaded polymersomes. A human breast cancer cell line (MCF-7) and human dermal fibroblasts, which served as the control cell line, were treated with free drugs, dual-drug-loaded polymersomes, or blank polymersomes to compare and assess their cytotoxicity. The slight cytotoxicity of nisin and CUR-loaded polymersomes to normal cells resulted in significantly greater inhibitory effects on cancer cells than did free drugs. Apoptosis assays and cellular uptake studies have also confirmed the findings derived from cytotoxicity studies (Salehi et al. 2024). Polymersomes could serve as a platform for encapsulating naturally occurring compounds with therapeutic effects to overcome their limitations (poor solubility and instability) and maximize their therapeutic effects by modifying their pharmacokinetics, enhancing their stability, and facilitating their co-loading with other therapeutic agents.

Tetrandrine is an alkaloid and a calcium channel blocker. It was isolated from Stephania tetransra. These findings indicate that tetrandrine inhibits proliferation and induces apoptosis in cancer and primary cancer cells, making it a potent candidate for cancer treatment. A recent study designed an antitumor agent of lactoferrin-conjugated biodegradable polymersomes to deliver DOX and tetrandrine for more effective chemotherapy of glioma. mPEG-PCL and R-carboxyl PEG-PCL were constructed as double-drug carriers to deliver DOX and tetrandrine simultaneously. To transport polymersomes across the blood‒brain barrier (BBB) and specifically target brain tumors, lactoferrin was conjugated as a glioma-specific ligand onto the surface of the polymersomes. *In vivo* imaging of glioma-bearing rats revealed that NIR dye-labeled lactoferrin-conjugated polymersomes successfully crossed the BBB and reached the tumor site. The tumor volume was significantly lower in the mice receiving lactoferrin-conjugated DOX and tetrandrine-loaded polymersomes compared to other treatments, and the median survival time of the former was higher than that of the other groups (Pang et al. 2010).

In a study, PCL-PVA was prepared sequentially by the preparation of hydrazine-modified PVA and aldehyde-modified PCL. The link between PCL and PVA was a pH-sensitive hydrazone bond. In the next stage, CUR and methotrexate were encapsulated within the prepared pH-sensing polymersomes. The CUR and methotrexate release tests revealed that higher amounts of the drug were released under acidic pH conditions, accounting for the system's pH sensitivity. A cytotoxicity assay was also conducted, and the findings revealed that the two drug-loaded polymersomes are more cytotoxic to MCF-7 cells than free individual drugs and single drug-loaded carriers. Additionally, the two drug-loaded polymersomes exhibited high cell uptake and were specifically targeted (Rahmani et al. 2023). The coencapsulation of CUR with methotrexate could provide a lower dosage needed for therapeutic effectiveness of the platform. On the other hand, the performance of the carrier in these types of platforms is crucial for achieving controlled on-demand release of the therapeutic agent and adjusting the ratio between therapeutic agents.

**Figure 5.**
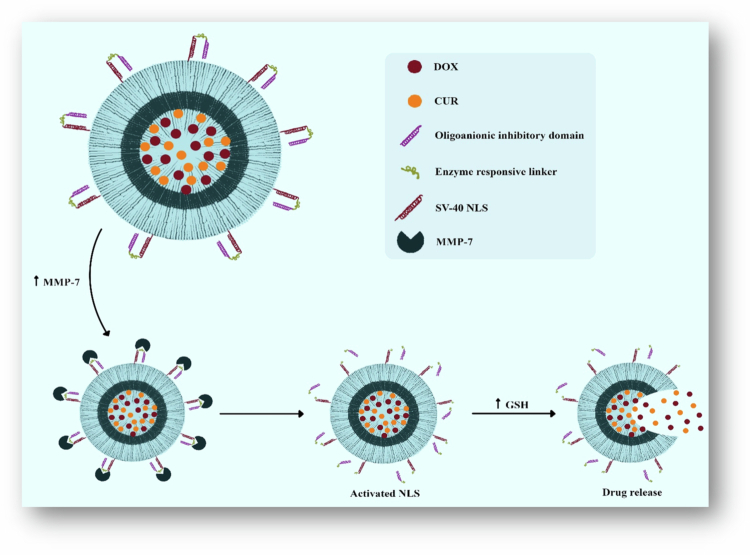
Illustration of redox-responsive polymersomes with masked NLS peptides for targeted delivery of DOX and CUR to pancreatic cancer microtumors. The figure was redrawn by the authors using Adobe Photoshop, based on information from Anajafi et al. (2017).

Nanovehicles decorated with nuclear localization peptide sequences (NLS) are carried into the cell nucleus. The nuclear localization peptides, nevertheless, are not selective and cannot distinguish between malignant cells and their normal counterparts. In this context, researchers designed a ‘masked’ NLS peptide that is only activated in the presence of overexpressed matrix metalloproteinase-7 (MMP-7) in the tumor microenvironment. In one study, a polymersome based on PEG (2000)-S-S-PLA (6100) was prepared for coencapsulation of CUR and DOX. The surface of this redox-sensitive polymersome was modified with an NLS and masked with the MMP-7 peptide. This advanced method aims to minimize off-target effects by utilizing mechanism-based drug release that targets the tumor microenvironment explicitly rather than normal tissues. The results from two-dimensional and three-dimensional culture models indicated highly inhibited growth of spheroids. Interestingly, polymersomes with both DOX and CUR acted as more effective therapeutics; in such cases, as many as 49% of the cancer spheroids were found to decrease in size, while only a 24% reduction was reported for normal cells (Figure 5) (Anajafi et al. 2017).

Another promising strategy for cancer treatment was explored using pH-sensitive polymersomes formulated from poly(2-(methacryloyloxy)ethyl phosphorylcholine) (PMPC) and poly(2-(diisopropylamino)ethyl methacrylate) (PDPA), which would allow for targeted drug delivery in head and neck squamous cell carcinoma (HNSCC). Hydrophilic DOX and hydrophobic PTX chemotherapeutic drugs, separately or in combination, can be encapsulated within polymersomes with high encapsulation efficiency and stability. The PMPC‒PDPA polymersomes are thus shown here to have preferential uptake in HNSCC cells compared to standard oral cells. Preferential uptake has been attributed to the increased expression of cancer cell class B scavenger receptors, specifically SR-BI. This finding reveals the potential of polymersomes to utilize receptor-mediated endocytosis to deliver drugs selectively and, consequently, have fewer side effects on normal tissues. The PMPC-PDPA polymersomes demonstrated the ability to penetrate deep within the hypoxic center of the spheroids over time, thereby overcoming the diffusion constraints commonly faced by traditional drug delivery systems. This deep penetration caused widespread cytotoxicity throughout the tumor model, ultimately compromising the structural integrity of the spheroids through prolonged exposure to drug-loaded polymersomes (Colley et al. 2014).

Autophagy is a controlled, destructive process, a natural mechanism by which dysfunctional or unwanted cellular components are broken down. Autophagy has now been implicated as a mechanism of protection of tumors against chemotherapeutic drugs, which also contributes to MDR. Although chloroquine phosphate (CQ) is an antiquated drug used for the treatment or prevention of malaria, recent work has revealed that CQ can inhibit Taxol transport mediated by *P*-gp in a dose-dependent manner and act as an autophagy inhibitor (Amaravadi et al. 2011).

In this study, the authors examined the effectiveness of polyphosphazene vesicles in delivering both DOX and chloroquine phosphate, a drug resistance-reversal agent, with the aim of enhancing anticancer activity via mechanisms that reverse drug resistance. This study highlights the self-assembly characteristics of amphiphilic polyphosphazenes (PEG/ethyl-*p*-aminobenzoate (EAB)-g-polyphosphazene), which enable the production of versatile new polymersomes capable of encapsulating DOX and chloroquine phosphate at a 1:1 or 2:1 weight ratio. The results revealed that the dual-drug-loaded polymersomes are highly cytotoxic to drug-resistant cancer cell lines, including adriamycin-resistant MCF-7 breast cancer cells and adriamycin-resistant HL60 leukemia cells. In vivo, a study using a zebrafish xenograft model demonstrated the enhanced antitumor efficacy of the designed polymersomes, which sensitized adriamycin-resistant MCF-7 cells to chemotherapeutic drugs (Xu et al. 2016). These results suggest that this codelivery strategy of DOX and chloroquine phosphate via polyphosphazene polymersomes may effectively eliminate MDR in cancer therapy, thereby improving the therapeutic efficacy of DOX in the clinic. Interestingly, high encapsulation efficiency of drug loading and amphiphilic polyphosphazene self-assembly into nanovesicles was achieved using a one-step dialysis process without the need for additional additives. Owing to the improved permeability and retention (EPR) effect of nanoscale polymersomes, coencapsulated drugs can accumulate in tumor tissue, providing a high therapeutic index.

In a different study, researchers developed a new polysaccharide polymersome system capable of targeting and delivering the two most prevalent anticancer drugs, DOX and cisplatin, to cancer cell sites. The polymersomes were formulated on a derivative dextran (DEX) backbone, which was elegantly designed by incorporating tetraphenylethylene (TPE), an aggregation-induced emission fluorescent material, and 3-pentadecylphenol (PDP), which facilitates the self-assembly of polymers into stable vesicles. This new architecture enables polymersomes to encapsulate drugs and ‘report’ their trajectory within cells in real time using fluorescence imaging. The resulting polymersome, DEX–PDP–TPE, offers a modular drug delivery platform. The most fascinating aspect of this study was possibly what happened to the polymersomes inside the cells. The fluorescence of the polymersomes was initially quenched because of energy transfer from the TPE chromophore to DOX. However, once within the lysosomes of the cancer cells, the enzymes trigger a burst of the polymersomes, releasing the drug and recovering its fluorescence. With this ‘turn-on’ fluorescent signal, researchers were able to monitor drug release into the cell nucleus and investigate the cytoplasmic location of the polymersomes. Compared with free cisplatin, cisplatin-conjugated polymersomes exhibited a significantly greater cancer cell-killing capacity, resulting in greater than 90% cell killing in MCF-7 breast cancer cells. Live-cell imaging revealed the stepwise breakdown of the polymersomes and the targeted intracellular delivery of DOX into the nucleus, providing important insight into the system's mechanism. This study is essential and represents a significant breakthrough in cancer nanomedicine. Not only do the DEX–PDP–TPE polymersomes deliver multiple drugs, but they also enable real-time observation of their movement through cells. As both therapeutic and bioimaging agents, these polymersomes are excellent tools for future innovations in cancer treatment (Virmani et al. 2021).

However, despite some successful examples, combining nanoparticles with drugs still has unpredictable results when moving from the laboratory to the clinic (Anselmo and Mitragotri 2016; Hua et al. 2018). This highlights the need to rethink how we develop drug combinations in the laboratory before they are translated to the clinic. Currently, we optimize drug combinations extensively in the laboratory before testing them in animals, relying heavily on measures such as drug IC50 values and combination indices to gauge success. Identifying other laboratory parameters that can help better predict how the treatment will work in patients remains a largely unexplored area, despite its potential to improve the translation of early-stage therapeutics to the clinic.

### 3.2. Combination of chemo-antiangiogenic therapy

The development of new vessels, or angiogenesis, is an active, multistep process that is regulated by a range of pro- and antiangiogenic drugs.

This process significantly influences tumor growth, invasion, and metastasis. Growth factors, chemokines, and adhesion factors are among the principal biomolecules involved in tumor angiogenesis and have been increasingly described and clarified with new developments in cellular and molecular biology. The development of antiangiogenic drugs as a result of targeted therapeutic research as a result of these molecules has made them options for antitumor therapy. Monoclonal antibodies and tyrosine kinase inhibitors (TKIs) targeting the vascular endothelial growth factor (VEGF) pathway are among the most widely used antiangiogenic drugs (Liu et al. 2023). According to Folkman's (1971) hypothesis, cancer cells are deprived of oxygen and nutrients if existing blood vessels are destroyed. New tumors did not develop; therefore, tumor growth is inhibited. His hypothesis was verified years later when the FDA approved bevacizumab, the first antibody against VEGF, as a cancer therapy (Lopes-Coelho et al. 2021).

A study examined the impact of co-loading CUR and angiostatin in PEG–PCL polymersomes as antiangiogenic therapy. Under physiological conditions (pH 7.4), the double-drug-loaded polymersomes exhibited controlled release of angiostatin and CUR, as confirmed by *in vitro* drug release profiles. Additionally, double-drug-loaded polymersomes were successfully delivered to the nucleus and cytoplasm of a human microvascular endothelial cell line (HMEC-1) and released within the cells, as indicated by confocal laser scanning microscopy and flow cytometry. Additionally, the prepared polymersomes suppressed vessel sprouting in the chicken chorioallantoic membrane in a chick chorioallantoic membrane assay (Cao et al. 2016).

In an attempt, biodegradable polymersomes were formulated for the combined delivery of an antiangiogenic agent, combretastatin-A4 phosphate (CA4P), and DOX to induce destruction of the tumor neovasculature and suppress cancer cell growth, thereby achieving synergistic anticancer activity. In this context, mPEG-PLA block copolymers were used as drug vehicles to formulate polymersomes containing DOX and CA4P (1:10). This polymersome exhibited strong, synergistic cytotoxicity against human nasopharyngeal epidermoid carcinoma (KB) cells. Moreover, it also accumulated at high levels in the KB tissue xenografts of BALB/c nude mice. As mentioned, the constructed polymersomes demonstrated significant anticancer activity *in vivo*, characterized by rapid destruction of tumor vascularization and long-term inhibition of tumor cell proliferation. (Zhu et al. 2015). The same team conducted another study in which the effects of engineered polymersomes on the sensitization of drug-resistant cancer cells to DOX were compared. A comparative approach was established to evaluate the efficacy of the inherent capacity of CA4P through the use of free drug and double-drug-loaded polymersomes. A 2D adriamycin-resistant MCF-7 cell culture model and 3D multicellular tumor spheroids were used in this study. These findings indicate increased DOX uptake, cytotoxicity, and apoptotic induction in adriamycin-resistant MCF-7 cells and adriamycin-resistant MCF-7 tumor spheroids after treatment with the fabricated polymersomes. The following possible molecular mechanisms are responsible for CA4P in addition to DOX resistance: inhibition of the activity of *P*-gp due to downregulation of the protein kinase Ca, augmentation of ATPase function, decreased ATP content, and increased intracellular levels of ROS (Zhu et al. 2017).

Traditionally, the toxicity-defined approach to drug dose development, often used for cancer chemotherapeutics, might not be suitable for antiangiogenic drugs. For instance, endostatin shows a U-shaped dose‒response curve, with the standard maximum tolerated dose (MTD) proving ineffective (Tjin Tham Sjin et al. 2006). When antiangiogenic drugs are combined with chemotherapy, antiangiogenic agents can normalize tumor blood vessels, increasing the effectiveness of chemotherapy. However, excessive inhibition of the tumor vasculature may reduce the penetration of the coadministered chemotherapeutic drug. Therefore, surrogate markers are crucial for monitoring antiangiogenic activity and could help predict a patient’s response to treatment (Jubb et al. 2006).

However, some poorly vascularized tumors can still respond well to treatments that block the growth of new blood vessels, as shown in several preclinical studies (Beecken et al. 2001; Fenton et al. 2004). It is possible that treating these poorly vascularized tumors with antiangiogenic drugs could reduce the already low blood supply to the point where the tumor can no longer grow. More research is needed to determine whether tumor microvessel density is a reliable indicator of how a tumor will respond to antiangiogenic treatment. Additionally, changes in tumor cell density after treatment may increase the difficulty of interpreting tumor microvessel density measurements (Hlatky et al. 2002; Ma and Waxman 2008).

### 3.3. Combination of chemo-radiotherapy

The therapeutic potential of X-rays, discovered by Wilhelm Conrad Röntgen in 1895, was first realized in cancer management. It has also been a century since Marie Curie received her second Nobel Prize for her pioneering research on radium, solidifying her position as a pioneer in radiation therapy. To commemorate this milestone, the year 2011 was regarded as the year of radiation therapy in the United Kingdom, with 100 years of achievement in the history of radiation therapy. From that momentous event forward, radiation therapy has become an established medical specialty, and radiation oncology has emerged as a branch of medicine comprising an alliance of health and science professionals from diverse areas of study. With surgery and chemotherapy, radiation therapy is a valuable and cost-saving cancer treatment option, representing only approximately 5% of the overall expenses of cancer treatment (Ringborg et al. 2003).

Chemoradiotherapy (CRT) involves the combined use of chemotherapy and radiotherapy in cancer treatment. Over time, it has become a standard approach for most types of locally advanced solid tumors. The foundation of CRT is based on the principles of spatial and in-field cooperation, which creates synergistic effects that boost the antitumor response by integrating both treatment methods. CRT is also associated with impressive patient survival rates and effective local disease control without causing severe long-term side effects. Although increased cytotoxicity may result in more damage to normal and cancerous tissues, CRT remains beneficial as long as its toxicity to normal tissues is less than that to tumor cells. Therefore, there is strong interest in optimizing CRT dosing, scheduling, and fractionation to maximize therapeutic benefits (Rallis et al. 2021).

Although combining systemic therapies and radiotherapy has shown limited success so far, these treatments can work synergistically in several ways that benefit each other. For example, radiotherapy can increase tumor sensitivity to treatment, while systemic therapies can help protect healthy tissue. Additionally, radiotherapy can target local disease, and systemic therapies can address distant micrometastases. This combination can also enhance the immune response and lead to increased cell death without increasing side effects (Zumsteg et al. 2024). Clinical trials have demonstrated that combining systemic therapies such as chemotherapy or androgen deprivation therapy with radiotherapy can improve survival in specific cancers, such as head and neck cancer, cervical cancer, lung cancer, and prostate cancer (Kishan et al. 2022; Lacas et al. 2021). These findings provide strong evidence that radiotherapy and systemic therapies can work together effectively. As radiation delivery has improved and more drugs have become available, new combinations of radiotherapy and systemic therapies are now ideal.

A study by He et al. outlined the creation of a pH-responsive polymersome platform designed for targeted delivery of glioblastoma multiforme therapy. The polymersomes were fabricated by self-assembling amphiphilic block copolymers, such as PCL-poly(2-ethyl-2-oxazoline) (PCL-PEOz), into stable pH-sensitive vesicular structures. This sensitivity enables the controlled release of drug-loaded therapeutic agents depending on the acidic microenvironment characteristic of solid tumors to improve drug bioavailability at the target site. The system is integrated with gold nanoparticles (AuNPs) for use as radiosensitizers, enhancing the efficacy of radiotherapy through improved tumor targeting and providing more therapeutically effective outcomes. Additionally, DOX is also loaded into the polymersomes, and the simultaneous delivery of AuNPs and DOX exerts a synergistic effect, significantly increasing antitumor efficacy against glioblastoma cells. Furthermore, Angiopep-2, a Kunitz domain peptide of aprotinin, is used to decorate the surface of polymersomes, targeting low-density lipoprotein receptor-related protein 1 (LRP1). This receptor is highly expressed in glioblastoma and brain endothelial cells, enhancing the targeting efficiency of therapeutic agents to cancer cells. Both *in vivo* and *in vitro* studies demonstrate that Angiopep-2 (ANG)-conjugated pH-sensitive polymersomes, Au-DOX@PO-ANG, significantly decrease tumor volume and prolong survival time in U87-MG human glioblastoma xenografts in BALB/c nude mice. These findings highlight the effectiveness of this system as a potential delivery vehicle for combination therapy, indicating its superior biocompatibility and low toxicity, which suggests its potential for application in clinical situations for glioblastoma treatment (He et al. 2021).

**Figure 6.**
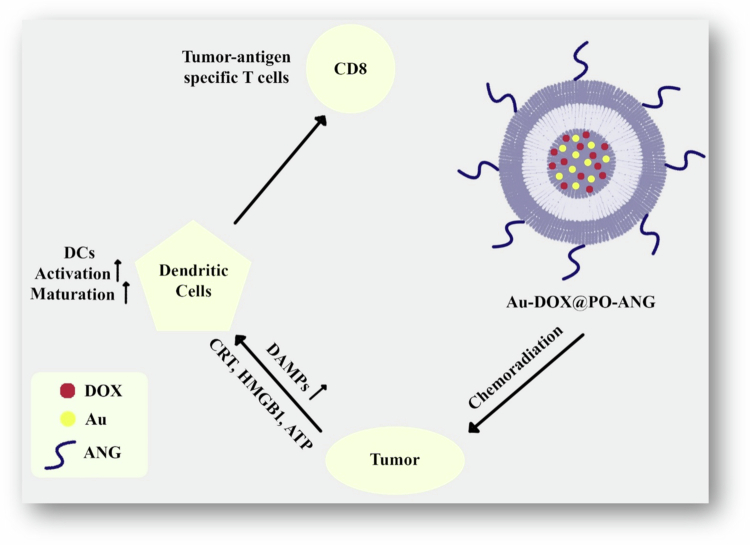
ICD induced by chemoradiotherapy of Au-DOX@PO-ANG. The figure was redrawn by the authors using Adobe Photoshop, based on information from He et al. (2021).

Cell death in cancer cells may be either immunogenic or nonimmunogenic, depending on the stimulus. Immunogenic cell death (ICD) is characterized by modifications of the cell surface composition and the release of soluble mediators, which are carried out in a well-defined temporal order. These signals activate various receptors on dendritic cells, thereby enabling the presentation of tumor antigens to T cells (Kroemer et al. 2013). In a different study, He et al. also assessed the immunogenic cell death caused by engineered polymersomes. They established an optimal approach to evaluate the capacity of chemotherapy and radiotherapy to induce immunogenic cell death and cancer cell death triggered by Au-DOX@PO-ANG + radiotherapy. It was shown that Au-DOX@PO-ANG + radiotherapy can induce calreticulin translocation to the cell membrane, HMGB1 and ATP release, and the maturation of bone marrow-derived dendritic cells. The practical vaccination efficacy of immunogenic cell death *in vivo* was demonstrated by the use of dying cancer cells from Au-DOX@PO-ANG combined with radiotherapy (Figure 6) (He et al. 2021).

### 3.4. Combination of chemo-magnetic hyperthermia

Since the 1950s, the concept of using magnetic implants for cancer therapy has been introduced, and such implants have been thought to have the ability to be thermoseeds, which generate heat under an alternating magnetic field. Magnetic nanoparticle-induced magnetic hyperthermia is a recent cancer therapy technique that leverages magnetic NPs' capacity to produce heat when subjected to an alternating magnetic field, thereby targeting the destruction of cancer cells. This method has several theoretical advantages, including the ability to treat deeply located tumors that are usually difficult to treat, the capacity to deliver treatment to a focused area with minimal damage to nearby healthy tissues, and the potential to explore combination therapies with other medicines to achieve the maximum overall effect (Vilas-Boas et al. 2020).

A research investigation aimed to synthesize DOX-encapsulated magnetic polymersomes as theranostic nanocarriers for use in magnetic resonance imaging (MRI) and magnetochemotherapy. The synthesis begins with a block copolymer, poly(trimethylene carbonate)-b- poly(L-glutamic acid) (PTMC-b-PGA), which is initially dissolved in dimethyl sulfoxide (DMSO). Water is then added slowly to enable nanoprecipitation. To impart magnetic behavior, hydrophobically modified ultrasmall superparamagnetic iron oxide nanoparticles, specifically maghemite (*γ*-Fe_2_O_3_), are incorporated into the DMSO solution before the addition of water. The weight ratio of the magnetic nanoparticles is precisely controlled, with a high loading of up to 70 wt% ultrasmall superparamagnetic iron oxide (USPIO) within the vesicle membrane without disrupting the structural integrity of the polymersomes. Co-loading of DOX and USPIOs is achieved by adjusting the DOX concentration during nanoprecipitation, thereby optimizing the drug loading efficiency. The resulting magnetic polymersomes are superparamagnetic and can be manipulated in an external magnetic field, increasing their efficacy in targeted drug delivery and MRI contrast. This study demonstrated that under hyperthermia induced by an alternating radiofrequency magnetic field, the release of DOX can be effectively regulated, further highlighting the multifunctional potential of these nanovehicles for cancer treatment (Sanson et al. 2011).

A new cancer therapy method was identified in a recent study, which created a multifunctional polymersome system capable of delivering targeted MRI contrast agents and chemotherapeutic drugs. Magnetic polymersomes encapsulated with DOX were synthesized via self-assembly of a copolymer infused with lipids, i.e., poly(acrylic acid-co-distearin acrylate) (poly(AAc-co-DSA)) in an aqueous dispersion of citric acid-coated superparamagnetic iron oxide nanoparticles (SPIONs). DOX was subsequently encapsulated via electrostatic interactions. For additional stability and targeting capacity, the polymersomes were further coated with chitosan and decorated with folate-conjugated poly(*γ*-glutamic acid-co-*γ*-glutamyl oxysuccinimide)-g-PEG to yield nanogel-caged polymersomes. The polymersomes exhibited great resistance to dilution and protein adsorption, and their drug release could be triggered externally via pH and temperature changes. Notably, when exposed to high-frequency magnetic fields, the nanogel-entrapped polymersomes showed greater cytotoxicity against HeLa cells compared to free DOX, confirming the efficacy of this combination therapy. Additionally, the SPIONs embedded within the polymersomes provided better r2 relaxivity than did the commercial SPION-based T2 contrast agent Resovist, thereby efficiently enhancing the MRI contrast of the targeted cancer cells. This research highlights the potential of folate-functionalized SPION/DOX-loaded nanogel-caged polymersomes as sophisticated theranostic nanodevices that integrate healing and diagnostic approaches to increase anticancer efficacy and decrease side effects. The construction of this novel system holds much promise for nanomedicine as a means to incorporate various modalities for superior cancer treatment (Chiang et al. 2013)

In another attempt, a diblock copolymer called PCL-s-s-poly(methacrylic acid) (PCL-s-s-PMAA) was synthesized, featuring a disulfide bond that connects its hydrophobic and hydrophilic blocks. The new copolymer was used to construct polymersomes that encapsulated truncated octahedral Fe_3_O_4_ NPs and DOX into a composite NP. Research has demonstrated the superparamagnetic nature of these NPs, which enables efficient magnetic targeting in the presence of an alternating magnetic field (AMF). When exposed to AMF, the temperature of the NPs increased considerably, reaching up to 54 °C within 15 min, thereby inducing hyperthermia and facilitating the rapid release of DOX in conditions with high GSH levels. This dual-responsive mechanism is particularly useful, as the GSH concentration in cancer cells is significantly higher than that in normal cells, thereby allowing targeted drug release only at the site of the cancer. *In vivo* tests with A549 tumor-bearing nude BALB/c mice demonstrated comprehensive tumor inhibition and enhanced MRI contrast resulting from the magnetic targeting of the NPs at the tumor site. These findings indicate that the synthesized polymersomes are potential theranostic agents that integrate MRI capability with drug delivery, providing a multimodal approach to cancer therapy. This new method not only increases the efficacy of chemotherapy but also reduces the systemic side effects that have traditionally been associated with chemotherapy, making it attractive for clinical application in targeted cancer therapy (Tsai et al. 2020).

Using magnetic hyperthermia alone is not enough to treat multiple or metastatic tumors. Therefore, combination therapies involving magnetic hyperthermia are becoming more popular, especially those that combine chemotherapy or immunotherapy (Pan et al. 2020; Wu et al. 2019). These approaches not only allow for effective tumor removal with lower magnetic field strengths and reduced drug doses but also offer significant advantages in targeting metastatic and multiple tumors (Chao et al. 2019; Zhang et al. 2020). Magnetic nanoparticles are widely used to develop combination therapy platforms based on magnetic hyperthermia because of their controllable size and shape and biocompatibility (Soetaert et al. 2020). However, magnetic nanoparticles that depend on relaxation losses and hysteresis losses to generate heat still face limitations in reaching adequate temperatures during magnetic hyperthermia.

Table 1 presents the characteristic properties (size, polydispersity index (PDI), zeta potential, loading efficacy or capacity, and release) of various polymersomal nanocarriers employed in chemotherapy-based combination therapy.

**Table 1.** Characterization of different polymersome-based nanovehicles used in chemotherapy-based combination therapy.

			Characterization						
Polymer	Encapsulated moiety	Targeting moiety	Size (nm)	PDI	Zeta potential (mV)	Loading efficacy (LE) or loading capacity (LC) (%)	Release (%)	Description	Reference
PEG-PCL	DOX	_	254 ± 7.7 (in PEG_45-_PCL_44_ based polymersome)	0.1 to 0.25	−14.8 ± 0.4 (in PEG_45-_PCL_44_ based polymersome)	LE = 35 to 39	DOX release:12% to 25% at 37 °C in pH 5 after 5 h (PEG_45-_PCL_44_ > PEG_114-_PCL_98_ > PEG_114-_PCL_114_) VEM release: 25% to 38% at 37 °C at pH 5 after 5 h (PEG_45-_PCL_44_ > PEG_114-_PCL_98_ > PEG_114-_PCL_114_)	The polymersomes were fabricated according to the thin-film hydration technique.	D'Angelo et al. (2022)
								Higher values of LC and LE were noted for VEM. Because it is hydrophobic, the VEM tends to interact with the hydrophobic bilayer because of the energy cost associated with its interaction with water. In contrast, the encapsulation of DOX primarily depends on the capture of a pseudoaqueous phase within the vesicles, with no significant interactions between the copolymers and DOX. Consequently, the EE is primarily influenced by the initial concentration of the drug in the hydration solution, which could explain the higher LC and LE amounts observed for VEM.	
			270 ± 6.5 (in PEG_114-_PCL_98_ based polymersome)		−15.2 ± 0.5 (in PEG_114-_PCL_98_ based polymersome)				
			282 ± 9.0 (in PEG_114-_PCL_114_ based polymersome)		−18.4 ± 0.6(in PEG_114-_PCL_114_ based polymersome)				
	VEM					LE = 43 to 55			
mPEG-PCL-PGA	DOX	*_*	44.9 ± 0.5	0.09	N/A	LE = 59.89 ± 3.40	DOX release: approximately 30% at pH 5 after 24 h Verapamil release: 91.74% at pH 5 after 24 h	The dialysis procedure was used to prepare the formulated polymersomes.	Li et al. (2015)
	Verapamil					LE = 84.82 ± 9.53			
mPEG-PLA	DOX	Folate	209 ± 34	0.64 ± 0.03	-2.4 ± 0.6	LE = 27.4 ± 5.5	Calcein release: around 80% in the presence of 5 mM GSH after 10 min	Thin-film hydration and solvent-exchange techniques were used to produce polymersomes and the pH gradient approach was used to encapsulate gemcitabine and DOX. Less than 5% of the polymersome contents would be released throughout their steady circulation in the blood and extracellular regions. They quickly release the enclosed contents within the cell cytosol following endocytosis. There were some enhancements (5 to 10%) in release when a reducing agent and ultrasound were applied together.	Nahire et al.(2014)
	Gemcitabine					LE = 46.1 ± 9.1			
PEG-S-S-PLA	DOX	Acridine orange	242.1 ± 6.6	0.3 ± 0.02	N/A	N/A	Calcein release: 15% in the presence of 10 mM GSH and 55% in the presence of 50 mM GSH after 40 min	Using the solvent exchange approach, polymersomes were synthesized. To verify the presence of the acridine orange on the surface of the polymersomes, the fluorescence emission spectra (excitation: 450 nm) of polymersomes was monitored before and after the ‘Click’ reaction	Anajafi et al.(2016)
	Gemcitabine								
PCL-ss-PEG-ss-PCL	DOX	Folate	208.47 ± 1.21	0.087	−1.74 ± 0.10	LE: 18.52	DOX release: approximately 100% at pH 7.4 in the presence of 10 mM GSH after 20 d PTX release: about 80% at pH 7.4 PBS in the presence of 10 mM GSH after 20 d TQR release: approximately 100% at pH 7.4 in the presence of 10 mM GSH after 20 d	The thin-film re-hydration and sonication method was used to create the redox-responsive and drug co-delivery polymersomes.	Qin et al. (2018)
	PTX					LE: 96.48			
	TQR					LE: 84.53			
PEG-b-PLys-SS-PCL	DOX	_	256 ± 5.6	N/A	43.9 ± 1.61	LE = 72 ± 1.12	DOX release: greater than 95% in the presence of 10 mM GSH within 12 h CPT release: greater than 95% in the presence of 10 mM GSH within 12 h	PCL was selected as the biodegradable hydrophobic block, PLys was responsible for cell permeability, and PEG was selected as the hydrophilic portion to shield the nanocarrier from proteolytic digestion and to give it a stealth feature *in vivo*. Polymersomes loaded with DOX and CPT were made using the solvent casting technique.	Thambi et al. (2012)
	CPT					LE = 44 ± 1.01			
PEG-b-PMPMC-g-PTX	PTX	_	148.3	0.035	N/A	N/A	PTX release: approximately 50% with 10 mmol/L dithiothreitol (DTT) after 48 h DOX release: almost 100% with 10 mmol/L dithiothreitol (DTT) after 48 h	The dialysis procedure was used to generate the polymersomes. The CAC of PMP polymersomes was 2.8 mg/L, as determined by fluorescence spectra using pyrene as a probe.	Xu et al. (2017)
	DOX					LC = 11.7			
	TQR					LC could not be measured accurately			
TMOR-CDAN	OXA	*_*	≈220	≈0.36	≈−32	LE ≈ 78	OXA and rapamycin release: less than 12% of the drug was released in the simulated stomach conditions, more than 50% was released from the duodenum to the jejunum, and the rest (38%) were released in simulated conditions of the lower intestines	The formulated polymersomes were prepared via the solvent switch approach. Modified chitosan diacetate (CDA) enhances the solubility and stability of the polymer, allowing it to encapsulate both hydrophilic oxaliplatin and hydrophobic rapamycin effectively. The poor swelling behavior of TMOR-CDAN at lower pH may be the cause of the noted low proportion of medicines released under simulated gastric environments.	Wande et al.(2022)
	Rapamycin					LE ≈ 80			
methyl-PEG–PLA	DOX	TLA	119.5 ± 2.11	0.117 ± 0.019	−12.59 ± 0.27	LE = 70.6 ± 1.4	DOX release: 76.8% at pH 4.5 within 72 h OXA release: 84.5% at pH 4.5 within 72 h	Polymersomes were prepared using solvent evaporation method.	Liu et al.(2019)
	OXA					LE = 81.5 ± 1.33			
PEG-PLA/PEG-PBD	PTX	*_*	N/A	N/A	N/A	N/A	PTX release: 60% at neutral pH within 24 h DOX release: 80% at neutral pH within 24 h	Polymersomes were prepared using sonication, freeze-thaw cycles, and extrusion.	Ahmed et al.(2006)
	DOX					N/A			
FA-F127-PDLLAGA	PTX	Folate	34.7 ± 1.1 (size of blank FA-F127-PDLLAGA polymersome)	N/A	N/A	LE = 3.31 ± 0.60	PTX release: 11.6% at pH 7.4 after 120 h DOX release: 63.9% at pH 7.4 after 120 h	The nanoprecipitation approach was used to prepare formulated polymersomes. The poor release rate of PTX could be attributed to the strong hydrophobicity of the drug. PTX simultaneously bonded firmly to the hydrophobic bilayer and repelled the external hydrophilic layer.	Wu et al.(2023a)
	DOX					LE = 13.88 ± 1.59			
PGA-PEG-PGA	PTX	*_*	420	0.1	N/A	LC = 21	Pt release: 22% in the presence of ascorbic acid and 3% in the presence of the thermolysin enzyme after 48 h	After the triblock copolymer was deprotected, the two anticancer medications could be anchored to the carboxylic groups of the PGA blocks. The inability of thermolysin (34.6 kDa) to pierce the PEG layer restricted its proteolytic activity to release the medication because it is considerably greater than that of ascorbic acid (176.1 g/mol).	Callari et al. (2017)
	Pt					LC = 28			
PLA-HA	DOX	HA	293.9 ± 17.53	N/A	N/A	LE = 54.05	DOX release: approximately 70% after 200 h Mel release: approximately 70% after 200 h	Solvent evaporation was used to generate Mel-loaded polymersomes, and an ion gradient technique was used to load DOX into the polymersome. Because DOX and Mel are both hydrophilic, their drug release patterns were similar.	Han et al.(2023)
	Mel					LE = 84.24			
HA-b-PCL	DOX	HA and FOXM1 aptamer	190.6 ± 12.0	0.18 ± 0.04	- 46.9	LE = 71.4 ± 4.0	DOX release: almost 100% at pH 5.4 after 216 h CPT release: almost 100% at pH 5.4 after 216 h	Polymersomes were prepared via nano-precipitation method.	Shahriari et al.(2021)
									
	CPT					LE = 51.0 ± 2.8			
mPEG-*b*-PNIPAM-*b*-P(DEAEMA-*co*-BMA)	DOX	*-*	168.3 ± 4.5	0.18 ± 0.004	N/A	LE = 59.9	DOX: almost 70% at pH 6.0 and 37 ℃ after 96 h PTX: almost 75% at pH 6.0 and 37 ℃ after 96 h	The polymersomes were produced via the direct dissolution method. At pH 6.0, the polymer P(DEAEMA-co-BMA) swells, enabling the rapid release of drugs. The polymer's lower critical solution temperature is approximately 41 °C, and below this temperature, the mPEG-b-PNIPAM corona swells, causing fast release of DOX. At 45 °C, the PNIPAM segment collapsed, slowing the release of DOX to less than 50% within four days. PTX cannot be successfully encapsulated because of the hydrophilic nature of the PDEAEMA block at pH 6.0, leading to nearly complete release of PTX at all temperatures	Zhou et al. (2021)
	PTX					LE = 70.2			
PEG, decyl chain and cholesterol	DOX	–	133 ± 35.5	0.43 ± 0.14	-2.79 ± 1.22	81.5 ± 5.5%	DOX release: over 40% in PBS after 24 h 5-FU release: more than 65% in PBS after 24 h Leucovorin release: over 30% in PBS after 24 h	The reverse-phase evaporation technique was used to fabricate polymersomes. The release pattern for each component exhibits a zero-order rate during the first two hours.	Aibani et al. (2018)
	5-FU					74.5 ± 20.1%			
	Leucovorin					72.1 ± 0.7%			
PCL_8000_-PEG_8000_-PCL_8000_	DOX	Folate	218.3 ± 11.2	0.192 ± 0.010	-1.84 ± 0.15	LE:23.55 ± 0.95	DOX release: almost 90% at PH 5.5 and 7.4 after 40 days PTX release: 46.08% at pH 5.5 after 40 days	Formulated polymersomes were developed via the thin film rehydration method and remote loading protocols. The gradual and prolonged release of PTX from the polymersome is due to the strong hydrophobic contact between PTX and the hydrophobic PCL membrane of the polymersomes.	Zhu et al.(2017)
	PTX					LE: 89.39 ± 4.46			
PEG-ss-PLA	Docetaxel	Folate	251 ± 4	0.24 ± 0.03	−4.75 ± 1.02	LE = 44	Mocetinostat release: 85% in the presence of 5 mM GSH	Solvent-exchange technique was used to generate polymersomes.	Karandish et al. (2016)
	Mocetinostat		271 ± 3	0.2 ± 0.01	−8.06 ± 1.89	LE = 80			
PVA–PEG graft copolymer-b-PCL	CUR	_	183 ± 5.01	0.16	N/A	LE = 93	Curcumin release: 80% at pH 5.5 after 48 h Nisin release: 60% at pH 5.5 after 48 h	Fabricated polymersomes were prepared via the microfluidics method.	Salehi et al. (2024)
	Nisin					LE = 78			
mPEG-PCL and HOOC-PEG-PCL	DOX	Lactoferrin	around 220	N/A	-9 to -10	LE = above 96%	N/A	Developed polymersomes were generated using the thin film hydration method.	Pang et al.(2010)
	tetrandrine					N/A			
PCL-PVA	Methotrexate	*_*	220	0.15	N/A	LE = 60	Methotrexate release: 63% at pH 5.5 after 48 h CUR release: 91% at pH 5.5 after 48 h	The polymersomes were developed using the solvent switch approach. The surface of prepared polymersomes has a negative charge that keeps particles from aggregating together, but after a few days in various media, the negative charge slightly diminishes, leading to a minor increase in polymersome size.	Rahmani et al. (2023)
	CUR					LE = 92			
PEG-PLA	DOX	NLS peptide	142 ± 4 (cytosolic environment (10 mM GSH)) and 308 ± 143 (nuclear environment (50 mM GSH)	0.4 ± 0.03 (cytosolic environment) and 0.9 ± 0.08 (nuclear environment)	−16.6 ± 0.9	LE = 56	DOX release: up to 94% in the presence of the MMP-7 enzyme and 50 mM GSH after 80 min CUR release: 45% in the presence of the MMP-7 enzyme and 50 mM GSH after 80 min	The polymersomes were prepared using solvent exchange method. NLS peptide was conjugated to polymersomes surface using azide-alkyne cycloaddition reaction.	Anajafi et al.(2017)
	CUR					LE = 47			
PMPC-PDPA	DOX	*_*	224.5 ± 43.5	N/A	N/A	LE = 37.1 ± 13.5	N/A	The fabricated polymersomes were prepared using the thin film hydration method.	Colley et al. (2014)
	PTX					LE = 42.7 ± 10.2			
PEG/EAB-*g*-polyphosphazene	DOX	*_*	49.30 ± 2.80	N/A	N/A	LE = 97.48 ± 2.13	DOX release: more than 80% at pH 5.5 after 10 h Chloroquine release: more than 90% at pH 5.5 after 10 h	The prepared polymersomes were self-assembled through a one-step common dialysis method. The effective intermolecular interaction between the benzene ring of the hydrophobic EAB group and the anthracene ring of DOX facilitated drug loading into the polymersomes.	Xu et al. (2016)
	Chloroquine					LE = 95.42 ± 4.37			
DEX–PDP–TPE	DOX	*_*	220 ± 20	N/A	N/A	LC = 3.8	DOX release: 95% at pH 7.4 in the presence of 10 U esterase enzyme after 48 h Cisplatin release: almost 90% at pH 7.4 in the presence of 10 U esterase enzyme after 48 h	Polymersomes were prepared using the dialysis method. Ester bonds were used to conjugate dextran with renewable PDP and TPE. Cisplatin was chemically attached to a carboxylic acid-functionalized version of the polymer (DEX–PDP–TPE–COOH) through a stable ester bond.	Virmani et al.(2021)
	Cisplatin		210 ± 15			LC = 11.5			
PEG–PCL	CUR	_	153	N/A	- 0.22	LE = 54.2	CUR release: 67.9% at pH 7.4 after 72 h Angiostatin release: 62.4% at pH 7.4 after 72 h	The film hydration approach was used to load angiostatin onto the polymersomes, and the soaking method was used to load CUR. Near-neutral zeta potential of polymersomes enhances the stability of drug formulations and extends their circulation time in the bloodstream.	Cao et al.(2016)
	Angiostatin					LE = 63.7			
mPEG-PLA	DOX	*_*	50.30 ± 3.78	0.222 ± 0.035	N/A	LE = 90.94 ± 1.32	DOX release: 90.76% at pH 5.5 after 72 h CA4P release: approximately 100% at pH 5.5 after 72 h	The formulated polymersomes were fabricated via solvent evaporation method.	Zhu et al. (2015), Zhu et al. (2017)
	CA4P					LE = 51.62 ± 1.10			
PCL-PEOz	AuNPs	Angiopep-2	210.8	N/A	N/A	LE of AuNPs-DOX = 89.5%	AuNPs-DOX release: 83.11 ± 3.18% at pH 5.5 after 96 h	The amino and carbonyl groups in the AuNPs and doxorubicin solution naturally reacted to form a conjugation. The thin-film rehydration technique was used to generate the pH-responsive polymersomes.	He et al. (2021)
	DOX								
PTMC-b-PGA	DOX	_	124 (in vesicles prepared in DMSO by adding water in 15 s loaded with 35% maghemite and 20% DOX feeding weight ratio, respectively) 61 (in vesicles prepared in DMSO by adding water in 5 s loaded with 50% maghemite and 20% DOX feeding weight ratio, respectively)	0.23 and 0.15	−39.3 and −42.0	LE = 52 and 70 (in vesicles prepared by adding water in 15 s and 5 s, respectively)	DOX release: approximately 31% at 23 °C with an oscillating magnetic field of frequency *f*_RF_ = 500 kHz and mean field intensity *H*_0_ = 2.12 kAm^−1^ after 7 h and approximately 50% at 37 °C in pH 7.4 after 24 h	The polymersomes were prepared via nanoprecipitation process. The polymersomes can respond to external stimuli, such as hyperthermia induced by an RF magnetic field, enabling controlled release of the encapsulated drug.	Sanson et al. (2011)
	Maghemite					N/A			
poly (AAc-co-DSA	DOX	Folate	221 (at pH 7.4) 243 (at pH 4.7)	0.13	≈−15	LE = 40.5	DOX release: more than 70% at pH 4.7 within 10 h	The particle size of SPION/DOX-loaded polymersomes rose slightly when the pH of the solution was changed from 7.4 to 4.7. The increased number of unionized *γ*-GA residues disrupted the chitosan/γ-PGA ionic complexes, and the increased protonation of the amino groups created an ionic osmotic pressure difference inside the interfacial polysaccharide layers, which was responsible for the swelling. When high-frequency magnetic fields caused hyperthermia at pH 4.7, the DOX fluorescence intensity of the SPION/DOX-loaded polymersomes increased by 100%. The improvement in drug release can be ascribed to the elevate in polymersome permeability, which is caused by the local temperature rising above the transition temperature of the hydrophobic lipid bilayer within vesicular membranes as a result of the magnetic hyperthermia from the embedded SPIONs under alternative magnetic fields.	Chiang et al.(2013)
	SPIONs					LE = 73.3			
PCL-SS-PMAA	DOX	*_*	210.4 ± 20.0 (size of blank polymersome)	0.150	−21.9	LE = 65	DOX release: 96% at pH 5.5 in the presence of 5 mM GSH with exposure to AMF at 10 kW for 15 min after 96 h	The double-emulsion approach was utilized in the formation of polymersomes.	Tsai et al.(2020)
	Fe_3_O_4_					N/A			

### 3.5. Combination of CDT and chemotherapy

In the past few years, Fenton and Fenton-like reaction-based chemodynamic therapy (CDT) has attracted much attention from researchers due to its excellent features, including high selectivity and localization, endogenous stimulation, and reduced multidrug resistance, as well as its superb efficacy as an antitumor treatment.

Although CDT shows excellent potential for treating tumors, its clinical use still faces several challenges. First, the relatively high pH of tumor sites does not favor most Fenton/Fenton-like reactions. Second, low concentrations of endogenous H_2_O_2_ in tumors cannot result in the production of continuous ROS, such as •OH. Third, high levels of reducing substances such as glutathione (GSH) in the tumor microenvironment weaken the therapeutic effect of CDT. These limitations make it difficult for CDT to completely eliminate malignant tumors on its own. As a result, many CDT-based combination treatment strategies have been developed. CDT-based combined therapy involves the integration of CDT with other cancer treatments, such as PDT, starvation therapy, and chemotherapy (Wang et al. 2020).

Regarding CDT-based combined therapy, cooperative increased interactions have been proven between several types of monotherapy using the combination of therapeutic agents within a single nanoplatform, which results in strikingly significant superadditive (i.e., ‘1 + 1>2’) therapeutic performances that are stronger than those of any monotherapy or their theoretical combinations.

Since CDT is hindered by the complexities of pathological microenvironments rich in GSH (which acts as antioxidants by reducing harmful hydroxyl radicals (OH•) through a process called scavenging), with diminished hydrogen peroxide (H_2_O_2_) levels and low pH levels that negatively affect its ability to achieve the best therapeutic outcomes and clinical translational applications, numerous groundbreaking designs of multi-configurable NPs have been explored to increase CDT efficacy over the years. The special characteristics of such platforms open new possibilities for the precise design and clinical application of CDT.

For this reason, numerous groundbreaking designs of multi-configurable NPs have been explored to increase CDT efficacy over the years. The special characteristics of such platforms open new possibilities for the precise design and clinical application of CDT.

One example is ferrocene (Fc)-based nanomedicines for enhanced chemodynamic therapy (ECDT), a new therapeutic approach that leverages the Fenton reaction. Owing to its good biocompatibility, potential in medicinal chemistry, and stable divalent iron ions, ferrocene is a popular choice as a Fenton's iron donor for tumor therapy. Ferrocene-based ECDT nanoplatforms have shown great promise for clinical use, significantly increasing the effectiveness of CDT treatment. These nanomedicines stand out for their exceptional consistency, low toxicity, high stability, and improved bioavailability, offering numerous benefits over traditional cancer treatments (Wu et al. 2025). Accordingly, some combinational platforms have been intelligently designed in order to improve CDT performance.

In this context, Wang et al. designed smart ferrocene-based responsive polymersome nanoreactors composed of self-assembling PEG-b-poly(2-(methacryloyloxy)ethylferrocene-carboxylate-co-2-(piperidin-1-yl)ethyl methacrylate) (PEG-b-P(FcMA-co-PEMA)) copolymers. These nanoreactors were formed by encapsulating glucose oxidase (GOx) and the hypoxia-activated prodrug tirapazamine within their inner aqueous compartments. PEG corona polymersome nanoreactors promote tumor accumulation upon intravenous injection into H22 tumor-bearing BALB/c mice. Piperidine protonation caused the PPEMA segments within the membranes to transition from hydrophobic to hydrophilic upon exposure to tumor acidity, thereby increasing the permeability of the nanoreactor membrane and facilitating the release of the loaded tirapazamine prodrugs. In polymersome nanoreactors, glucose and oxygen from the tumor can enter and be catalyzed by GOx to convert into H_2_O_2_, which is transformed into •OH by the Fenton reaction via ferrocene moieties. Oxygen consumption may exacerbate tumor hypoxia, triggering hypoxia-activated tirapazamine prodrugs that can generate •OH and benzotriazinyl radicals. In amplified CDT, all the generated radicals work together to suppress tumor growth and effectively kill tumor cells (Wang et al. 2021).

A highly promising cancer treatment is dynamic therapy, such as sonodynamic therapy (SDT), photodynamic therapy (PDT), or chemodynamic therapy (CDT) (Cui et al. 2021; Xu et al. 2021). Sensitive chemicals are employed in PDT and SDT techniques to catalyze the production of reactive oxygen species (ROS) in response to light or ultrasonic waves (Cui et al. 2021; Xu et al. 2021). CDT treatment does not require any external energy. CDT nanomaterials can transform existing H_2_O_2_ into ROS through catalysis or Fenton/Fenton-like reactions of dissociated transition metal ions (Cui et al. 2021). Fenton and Fenton-like reactions are well established as lethal producers of ROS, especially ·OH, in cancer treatment. In this sense, the Fenton and Fenton-like reactions can transform H_2_O_2_ into more hazardous **·**OH radicals. As active single-electron molecules, ·OHs have a strong oxidizing ability that damages DNA and oxidizes amino acid termini in proteins and polyunsaturated fatty acid chains in lipids. Ferroptosis, a type of cell death caused by oxidative membrane damage, results from this process. This phenomenon results from a decrease in glutathione peroxidase activity, an enzyme that plays a crucial role in repairing this type of damage (Mohammadi Zonouz et al. 2024; Nourollahian et al. 2024).

In a study of tumors, pH-responsive, selective, membrane-permeable therapeutic nanoreactors were synthesized to increase the activation and delivery of prodrugs for cancer therapy. This study aims to develop and utilize polymersome nanoreactors that can directly activate prodrugs at the tumor site into active chemotherapeutic species, thereby preventing the systemic toxicity typically associated with conventional chemotherapy. The researchers designed a diblock copolymer-based polymersome nanoreactor, namely, PEG-block-poly(benzyl methacrylate-co-(1-piperidinyl)ethyl methacrylate) (PEG-b-P(BzMA-co-MPE)). The hydrophobic anticancer prodrug investigated here was the CPT prodrug, which was encapsulated via a phenylboronic ester moiety to facilitate its subsequent activation. Hydrophilic GOx was also encapsulated within the polymersomes. When administered, the polymersome nanoreactor is inactive during its passage through the bloodstream and healthy tissues. Following its acidification to an acidic microenvironment characteristic of tumor tissues (pH 6.5–6.8), the permeability of the pH-sensitive membrane begins to increase, allowing small molecules such as glucose and oxygen to pass through the membrane into the interior and react with GOx to form H_2_O_2_. The generated H_2_O_2_ further triggers the cleavage of self-destructive phenylboronic ester groups, which accordingly initiates the release of the prodrug CPT's active species within the tumor microenvironment. The results revealed that the polymereome nanoreactors prepared in this work resulted in increased localized activation of the prodrug and significantly improved antitumor effectiveness as a result of the synergistic effects of oxidation and chemotherapy. *In vitro* and *in vivo* tests on H22 tumor-bearing ICR mice confirmed that a concurrent combination of prodrug activation and H_2_O_2_ resulted in potent tumor cell killing with reduced cytotoxicity toward normal tissues. The findings suggest that this broad-spectrum and potent prodrug delivery and activation approach for anticancer drugs may be extended to other agents, potentially revolutionizing targeted anticancer therapies with improved patient benefits (Mukerabigwi et al. 2019).

In an attempt to investigate the theranostic effect of cisplatin and indocyanine green (ICG)-loaded Fe_3_O_4_-embedded succinic peroxide-poly lactic-co-glycolic acid (Pt/Fe_3_O_4_@SP-PLGA) lipopolymersomes (LPs), a study was conducted in BALB/c nude mice with MCF-7 tumors. Lecithin and (PEG-PLGA)2-SP were utilized to synthesize LPs. Pt/Fe_3_O_4_@SP-PLGA LPs were made water-dispersible, proton-penetrable, and biocompatible by incorporating hydrophilic polymers containing grafted PEG units as surface capping molecules on the NPs. By regulating the reaction between SP and iron oxide, the therapeutic moiety •OH is produced in an acidic pH environment and under NIR irradiation. Owing to external NIR irradiation, the Pt/Fe_3_O_4_@SP-PLGA LPs quickly depolymerized into a progressively loose structure and further collapsed with increasing interior temperature. ^1^O_2_ species were also generated because of the reaction between released Fe^2+^ and dissociated SP in the H^+^ environment. Moreover, the encapsulation of cisplatin had the desired synergistic effect and improved the killing effect on tumor cells (You et al. 2019).

Thus, combining CDT with chemotherapy can lead to effective treatment results owing to a significant synergistic effect, with a wide range of potential applications.

### 3.6. Combination of CDT and starvation therapy

Owing to the high rate of metabolism and tumor growth, the majority of tumor cells transport and metabolize nutrients at a much higher rate than normal cells do. Therefore, following glucose deprivation, cancer cells are highly susceptible to variations in glucose concentrations (Shao et al. 2021). Most studies have used the GOx enzyme to oxidize glucose by catalyzing its reaction with gluconic acid and H_2_O_2_, thereby breaking it down. In addition to glucose-blockade-induced deprivation of energy, a surplus of H_2_O_2_ produces systemic toxicity, which increases the effectiveness of killing cancer cells (Yu et al. 2021).

**Figure 7.**
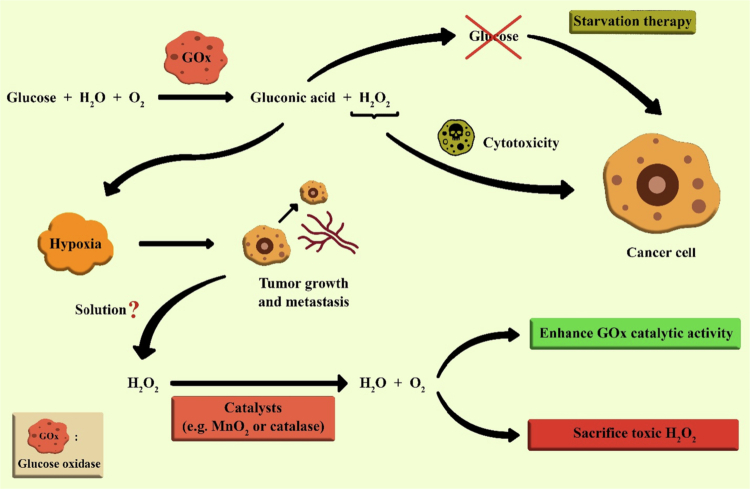
Schematic illustration of glucose oxidase (GOx)-mediated cancer starvation therapy. GOx catalyzes the oxidation of glucose into gluconic acid and hydrogen peroxide (H_2_O_2_), which results in glucose depletion and starves cancer cells. Additionally, the buildup of H_2_O_2_ has cytotoxic effects on tumor cells. However, this process consumes oxygen, worsening hypoxia in the tumor microenvironment, which can promote tumor growth and metastasis while reducing GOx activity. To overcome this issue, catalysts such as MnO_2_ or catalase can break down H_2_O_2_ into water and oxygen, increasing local oxygen levels and increasing GOx activity. However, this decomposition reduces H_2_O_2_'s toxic effects, potentially lowering treatment effectiveness. The original image was drawn via Adobe Photoshop.

Nevertheless, this process utilizes oxygen and exacerbates local hypoxia *in vivo*. As solid tumors are naturally hypoxic, the effectiveness of GOx-mediated cancer starvation therapy is compromised by an insufficient oxygen supply. GOx catalyzes glucose through an aerobic glycolysis pathway, and the consumed oxygen exacerbates hypoxia within the tumor (Figure 7). This not only alters enzymatic activity but also facilitates tumor growth and metastasis. Thus, numerous attempts have been made to improve the efficacy of GOx-mediated starvation therapy. For instance, H_2_O_2_ has been catalyzed into H_2_O and O_2_ using catalysts such as MnO_2_ and catalase, which increases the local oxygen pressure and therefore enhances the catalytic activity of GOx. Unfortunately, this strategy reduces treatment efficacy by sacrificing toxic H_2_O_2_. However, starvation therapy may be less efficient if substrates such as lactate, glutamine, and aspartate are generated from glucose starvation (Shao et al. 2021; Yu et al. 2021).

The following are the benefits of GOx-mediated tumor treatment (Shao et al. 2021):

1) GOx is a naturally occurring protein oxidoreductase that has excellent biocompatibility and biodegradability.
2) Gluconic acid production increases the acidity of the tumor microenvironment, which may lead to the release of drugs that respond to pH changes.
3) H_2_O_2_ generation increases the oxidative stress level in tumors, which may result in cell death.
4) GOx-mediated starvation therapy can be combined with other treatments to provide synergistic cancer therapies and increase the therapeutic outcome.

As previously reported, H_2_O_2_ produced in glucose oxidation induced by GOx can break down into highly toxic hydroxyl radicals through the Fenton reaction when combined with CDT agents. Then, platforms that can deliver both GOx and CDT agents together have shown great potential for cancer treatment (Yu et al. 2019).

**Figure 8.**
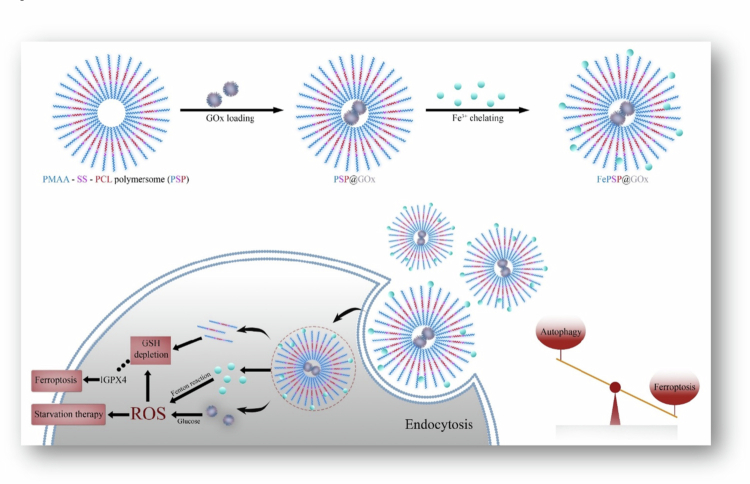
Illustration of the development of FePSP@GOx NPs and their antitumor effects through ferroptosis and starvation therapy. The figure was redrawn by the authors using Adobe Photoshop, based on information from Lo et al. (2024).

In this context, a ferric ion and GOx-filled polymersome-based nanoreactor was used in research to induce cascade reactions for cooperative and multifunctional anticancer therapies. A disulfide linkage at the junction between the hydrophobic and hydrophilic portions of the redox-sensitive PCL–SS–PMAA was used to synthesize the nanoreactor. The hydrophilic interior of the polymersome was encapsulated with the oxidoreductase enzyme to cause starvation, and the PMAA units forming the exterior shell were used to chelate the Fe^3+^ ions. By various mechanisms, the constructed polymersome showed the following actions: (1) the polymersome could establish a nanoreactor by double-loading with GOx and chelating with ferric ions; (2) the disulfide crosslinks in the polymersome were cleavable within the range of levels of GSH in tumor cells. The glucose present endogenously is oxidized by the released GOx to produce H_2_O_2_, which induces peroxide damage and starvation. Later, excess H_2_O_2_ depletes GSH, causing an intracellular redox imbalance. (3) The chelation of Fe^3+^ ions and the ROS-activated cascade reaction promote overactivated ferroptosis. When treated with prepared polymersomes, the MDA-MB-231 cell line exhibits effective depletion of glutathione peroxidase 4 (GPX4) and GSH. MDA-MB-231 cells also exhibited a high rate of lipid peroxidation after treatment with the formulated NPs. In the cytotoxicity test, MDA-MB-231 cells treated with the prepared polymersome exhibited a high degree of cell death. Overall, cell death is ultimately initiated by the dominance of the ferroptosis mechanism over autophagy, despite starvation therapy eliciting a protective autophagic response (Figure 8) (Lo et al. 2024).

### 3.7. Combination of CDT and PDT

PDT primarily depends on the generation of singlet oxygen, which is induced by the activation of a photosensitizer, enabling the destruction of targeted tumor cells. Therapy can be used in the treatment of various malignant diseases. Specifically, the initial preclinical applications extended back to the early 1900s, with the report of the application of PDT in managing skin tumors by Dougherty in 1978. Subsequent research in approximately 1980 again demonstrated the efficacy of PDT and encouraged additional researchers from a broad cross-section to become interested (Gunaydin et al. 2021). PDT has not yet achieved complete success in the clinical destruction of tumors. Several reasons have been proposed for this result. One hypothesis is that the greater the distance from the vascular supply is, the less effective PDT will be in inducing tumor cell death. In contrast, a heterogeneous distribution of the photosensitizer in the tumor tissue can have severe consequences. Therefore, various studies have aimed at developing different strategies, such as targeted delivery protocols in PDT and their combination with other treatment modalities.

Researchers have found that combining Fenton reaction-based CDT with PDT can effectively trigger tumor ferroptosis by generating various reactive oxygen species (ROS, including hydroxyl radicals, singlet oxygen, and superoxide), which then strongly suppress tumor growth (Chen et al. 2024).

In order to adapt to the distinct environment of solid tissues, tumor cells have developed various mechanisms to resist ferroptosis. These include increased glutathione (GSH) production, reduced iron accumulation, and the activation of other antioxidant defenses, all of which help tumor cells endure oxidative stress. Relying solely on increasing intracellular reactive oxygen species (ROS) through ferrous ions in chemodynamic therapy may not be sufficient for effective ferroptosis induction (Chen et al. 2024).

In this regard, in a study, cross-linked multifunctional polymersomes encapsulating the photosensitizer chlorin e6 and the hypoxia-activated prodrug tirapazamine coloaded with GSH triggered persistent GSH depletion for synergistic PDT and hypoxia-activated CDT in 4T1 tumor-bearing mice. Uncrosslinked polymersomes are prepared from a grafted polymer with the formulation poly(oligo(ethylene glycol)-co-4-(((4-nitrophenyl)carbamoyl)oxy) benzyl methacrylate) (POEG-PNPA). Upon reaction with GSH, decrosslinking is facilitated for on-demand drug release in the tumor microenvironment through the disulfide bonds of the polymersome. Moreover, GSH may be further depleted by the highly reactive quinone methide produced. Tumor oxidative stress increases due to GSH depletion, leading to enhanced chlorin e6-mediated PDT when tumors are irradiated with 660 nm light. The tirapazamine prodrug is activated by increased tumor hypoxia caused by PDT, thereby increasing the activity of PDT and hypoxia-activated CDT. Furthermore, the newly synthesized polymersome exhibited effective GSH depletion and high anticancer activity both *in vitro* and *in vivo* (Cheng et al. 2024).

### 3.8. Combination of chemo-PDT

In order to overcome the deficiency of traditional cancer therapy and enhance therapeutic efficiency, integrating PDT with chemotherapy could be a new avenue for treating cancer.

Researchers have been actively working on combining chemotherapy and PDT through the codelivery of both chemotherapeutic drugs and PSs. One approach is to combine both agents through chemical conjugation (Shiah et al. 2001). However, this method has a significant drawback: the drugs lose their effectiveness, as they are directly exposed to blood enzymes. To overcome this challenge, various types of nanoparticles have been used to prolong the circulation time and successfully deliver drugs (Lee et al. 2018). For instance, polymeric nanoparticles have shown promise.

Additionally, the use of nanoparticles as codelivery systems can be hindered by structural competition between the two drugs. As a result, to increase the therapeutic efficiency of combination therapies, we need a new approach to overcome the uneven delivery efficiency and low drug encapsulation efficiency by creating more versatile vehicles.

In this regard, a study synthesized a polypropylene sulfide (PPS)-b-PEG block copolymer, which was subsequently employed to form the polymersome architecture. Zinc phthalocyanine, a hydrophobic photosensitizer, was encapsulated in the polymersome shell, and DOX was loaded into the inner aqueous core. Upon irradiation of the zinc phthalocyanine with 660 nm NIR light, singlet oxygen ^1^O_2_ molecules were produced. These molecules oxidize the adjacent sulfur atoms at the PPS block, ultimately rupturing the entire polymersome structure and releasing the entrapped DOX. Under laser stimulation and drug release at the tumor sites, the released DOX and ^1^O_2_ could have an additive effect on cancer therapy. The NIR irradiation-induced production of singlet oxygen and release of DOX were verified using *in vitro* experiments. *In vivo* experiments have demonstrated that nanosystems combining PDT and chemotherapy effectively accumulate in A375 melanoma tumors of BALB/c nude mice, resulting in potent inhibition of tumor growth (Tang 2020).

**Figure 9.**
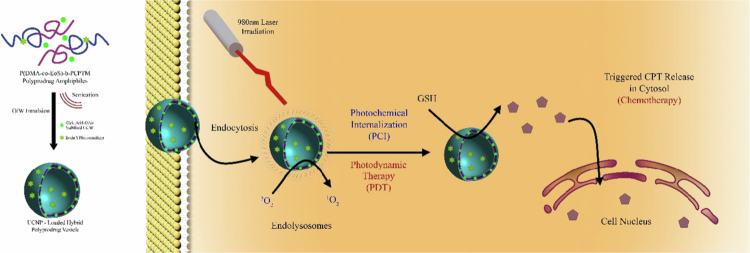
A schematic illustrating the fabrication process for UCNP-loaded hybrid vesicles. Upon exposure to a 980 nm laser, energy transfer occurs from the UCNPs to the EoS moieties inside the hybrid vesicles, activating EoS sensitizers and generating singlet oxygen (^1^O_2_). The produced ^1^O_2_ induces both PDT and PCI effects, facilitating the endosomal escape of hybrid vesicles into the cytosol. This process subsequently triggers the GSH-mediated release of CPT from the vesicle bilayers, leading to synergistic cancer cell eradication via the combined actions of PDT and chemotherapy. The figure was redrawn by the authors using Adobe Photoshop, based on information from Zhu et al. (2017).

In a different work, poly(*N*, *N*-dimethylacrylamide-co-eosin Y (EoS)-b-poly(CPT-based monomers (CPTM)) amphiphilic polyprodrugs were synthesized. The amphiphilic polyprodrugs formed hybrid vesicles through self-assembly with the help of an oil-in-water (O/W) emulsion process in the presence of hydrophobic oleic acid (OA)-stabilized upconversion NPs (UCNPs, NaYF4:Yb/Er). This facilitated EoS photosensitizer excitation by NIR laser irradiation, which produces ^1^O_2_ may not only induce a PDT effect but also destabilize endolysosome membranes, hence making it easier for endocytosed nanocarriers to escape the endosome. The hybrid vesicles can be easily internalized by endocytosis, as demonstrated by cell experiments on HepG2 cells. The internalization process of ^1^O_2_ caused instantaneous endosomal release within the cytoplasm under laser irradiation at 980 nm, although hybrid vesicles initially taken up localized within endolysosomes. The chemical action was then activated once the escaped hybrid vesicles induced GSH-facilitated release in the cytoplasm (Figure 9) (Zhu et al. 2017).

### 3.9. Combination of SDT and chemotherapy

Sonodynamic therapy (SDT) is a noninvasive approach for treating deep tumors and uses focused ultrasound (US) to activate sonosensitizers and generate reactive oxygen species (ROS) for localized cell damage (Padma 2015; Trendowski 2014; Wang et al. 2020). Unlike photodynamic therapy (PDT), which relies on light, SDT leverages the superior tissue penetration of US, enabling the treatment of deep-seated tumors without harming nearby healthy tissues (Lafond et al. 2019; Nguyen et al. 2020). However, the anticancer activity of SDT is currently limited by the poor delivery of sonosensitizers into cells. To make SDT more effective, it is essential to develop safe drug carriers that can enhance the cellular uptake and bioavailability of sonosensitizers. Using nanocarriers to deliver sonosensitizers has become a promising way to address the major drawbacks of sonosensitizers. Nanocarriers are key to improving the cellular uptake and availability of sonosensitizers (Kang et al. 2024).

In this regard, a study constructed PLA-PEG polymersomes with a high drug loading capability for verteporfin and DOX, enabling efficient combination therapy both *in vitro* and *in vivo*. The hydrophilic agent DOX was encapsulated within the hydrophilic core, whereas the hydrophobic agent verteporfin was encapsulated within the hydrophobic shell. Upon release of DOX, the sonodynamic activity of verteporfin results in the generation of ROS when the polymersomes are subjected to ultrasound treatment. The results of the cytotoxicity test revealed that the cellular uptake efficiency of the resulting polymersomes was greater than that of the single treatment. Moreover, *in vivo* biodistribution showed that verteporfin-DOX-polymersomes accumulated at MIA PaCa-2 tumor sites in BALB/c nude mice bearing tumors. Consequently, these polymersomes displayed enhanced anticancer activity compared to either of the monotherapies (Kim et al. 2023).

### 3.10. Combination of chemo-CDT-PDT

Chemotherapy is a common treatment option for cancer that has spread throughout the body. It may be used alone or in combination with surgery, PDT, CDT, or other therapies to effectively treat cancer. Cancer is a complex issue. In this context, the use of a multimodal treatment approach that combines several methods can produce the best results in cancer therapy. In this regard, chemotherapy plays a vital role, as it provides a powerful way to target cancer cells and enhance overall treatment success.

**Figure 10.**
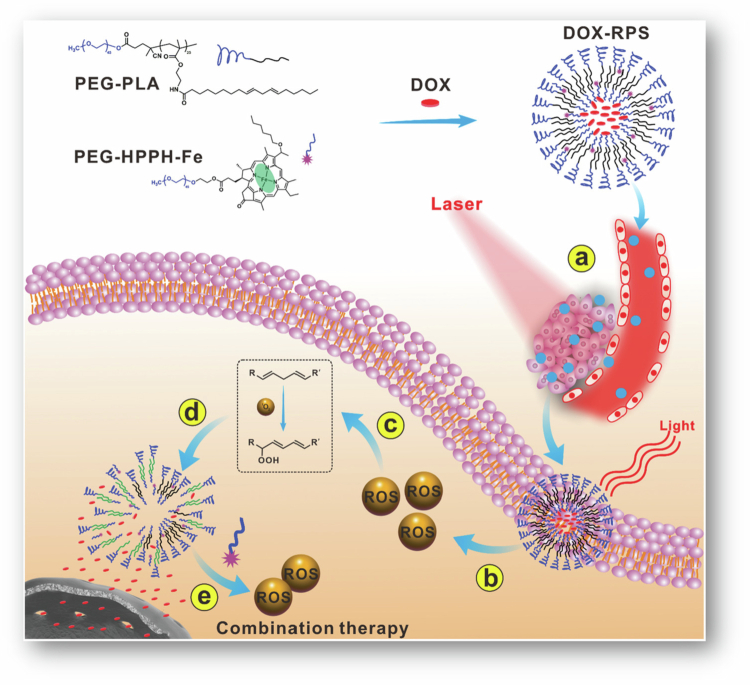
Representation of doxorubicin hydrochloride-encapsulated ROS-responsive polymersome (DOX-RPS) using photodynamic-chemodynamic cascade reactions for drug delivery and chemo-ROS dual therapy. (a) Through passive targeting, nanosized DOX-RPS accumulates in tumor tissue; (b) HPPH produces ROS via a photodynamic reaction during laser irradiation; (c) the ROS then oxidize linoleic acid to linoleic acid peroxide; (d) the generated LAP molecules alter the structure of the RPS, permitting drug release. (e) A Fenton-like reaction regenerates ROS when HPPH-Fe is present. The released medication and the generated ROS can result in improved combination therapy. Reprinted with permission from Wang et al. (2021).

An investigation utilized the amphiphilic self-assembly of PEG-poly(linoleic acid) and PEG-(2-(1-hexyloxyethyl)-2-devinyl pyropheophorbide-α)-iron chelate (PEG-HPPH Fe) to construct a DOX-loaded ROS-responsive polymersome. The drug can be transported successfully to the target tumor site using polymersomes, which employ a passive targeting effect. Following the accumulation of tumor tissue by the DOX-ROS-responsive polymersome, the photosensitizer HPPH effectively produces ROS after laser irradiation, resulting in the *in situ* peroxidation of the linoleic acid chain. The structural stability of the polymersome is impaired by the generated linoleic acid peroxide, allowing triggered drug release. A Fenton-like reaction is used to recycle ROS from linoleic acid peroxide, which is catalyzed by HPPH-Fe. Thus, it is possible to achieve ROS-induced drug release without excessive consumption of ROS. The potent anticancer activity, responsive drug release characteristics, and ROS generation of the formed polymersomes were shown *in vitro* and *in vivo* in U87MG tumor-bearing athymic nude mice. A potential strategy for optimizing combination therapy is the photodynamic-chemodynamic cascade system (Figure 10) (Wang et al. 2021).

### 3.11. Combination therapy using CDT, chemotherapy, and starvation therapy

Polymersomes could also provide a platform for cooperative cancer therapy, combining more than two treatment modalities.

In this regard, a PEG-b-*P*-(copolymerized block of thioketal-linked camptothecin methacrylate (CPTKMA))-co-PEMA) polymersome nanoreactor with tumor acidity-responsive membrane permeability was employed in a study to initiate cascade reactions for coordinated cooperative cancer therapy. The nanoreactors are responsive polyprodrug polymersomes with GOx and ultrasmall iron oxide NPs in the inner aqueous compartments and membranes, respectively. In particular, an acidic tumor microenvironment can trigger cascade reactions via the permeability of ultrasensitive pH-responsive membranes and the diffusion of small molecules. These are (i) GOx-catalyzed glucose oxidation resulting in the production of H_2_O_2_ and gluconic acid; (ii) acid-mediated release of iron ion (Fe (Khan et al. 2024)/^3+^); (iii) Fenton reaction between Fe (Khan et al. 2024)/^3+^ and H_2_O_2_ resulting in the production of •OH; and (iv) •OH-mediated release of CPT by thioketal linkers. Herein, the substances consumed *in situ* and those formed in situ are applied to enable efficient tumor inhibition during coordinated, cooperative cancer therapy, which consists of chemotherapy, CDT, and starvation therapy. Nanoreactors infused intravenously efficiently inhibited highly invasive H22 mouse hepatoma and A549 human lung carcinoma in murine cancer models, as revealed by antitumor evaluation (Ke et al. 2019).

This study clearly showed the versatility of the polymersomal structure for designing complex cancer treatment modalities with multipurpose characteristics.

Table 2 shows the characteristics (size, PDI, zeta potential, loading efficiency or capacity, and release) of different polymosomal nanovehicles used in dynamic therapy-based combination therapy.

**Table 2.** Characterization of different polymersome-based nanocarriers used in dynamic therapy-based combination therapy.

			Characterization						
Polymer	Encapsulated moiety	Targeting moiety	Size (nm)	PDI	Zeta potential (mV)	Loading efficacy (LE) or loading capacity (LC) (%)	Release (%)	Description	Reference
PEG-b-P(FcMA-co-PEMA)	Tirapazamine	_	97.9 (pH 7.4)	0.08	N/A	LC = 5.2	Tirapazamine release: more than 70% at pH 6.5 after 24 h	The polymersomes were constructed using the solvent replacement approach.	Cui et al.(2021)
	GOx		110.6 (pH 6.5)	0.17		LC = 6.4			
PEG-b-P(BzMA-co-MPE)	GOx	*_*	≈200	N/A	−4.83 (potential of blank polymersome)	LE = 12.86	CPT release: approximately 80% at pH 6.8 within 48 h	The formulated polymersomes were prepared via solvent replacement method. CPT prodrug was encapsulated in the hydrophobic membrane via *π*-*π* stacking and hydrophobic interactions.	Mukerabigwi et al.(2019)
	CPT prodrug					LE = 34.63			
(PEG-PLGA)_2_-SP	Cisplatin	_	113.6	0.226	≈−25	LC = 4.85	Cisplatin release: about 99.98% at pH 5.5 after NIR irradiation in 36 h Fe_3_O_4_ release: 89.68% at pH 5.5 after NIR irradiation in 36 h	Nanocarriers having w/o/w layered structures were generated by using the double emulsification method.	You et al. (2019)
	Fe_3_O_4_					LC = 9.81			
	Succinic peroxide					LC = 3.45			
PCL–SS–PMAA	GOx	*_*				= 8.4	N/A	The polymersomes were fabricated using the thin film hydration technique.	Lo et al. (2024)
POEG-PNPA	Chlorin e6	_	104 ± 10	N/A	−18.3 ± 2.3	LE = 87.5	Chlorin e6 release: 73% in the presence of 10 mM GSH within 12 h Tirapazamine release: 77.54% in the presence of 10 mM GSH within 12 h	Triethylamine and cystamine were added to the polymersome solution to generate crosslinked polymersomes.	Cheng et al. (2024)
	Tirapazamine					LE = 20.2			
PPS-b-PEG	DOX	_	≈150	N/A	N/A	LE = 41.01	DOX release: more than 50% after 660 nm laser irradiation after 24 h	The formulated polymersomes were generated by solvent dispersion method.	Tang(2020)
	Zinc phthalocyanine					LE = 69.92			
Poly(*N*,*N*-dimethylacrylamide-co-EoS)-b-PCPTM	CPT	*_*	136	0.069	N/A	LC = 51.6	CPT release: 89% in the presence of 10 mM GSH within 60 h	Solvent evaporation method was used in the development of hybrid vesicles.	Zhu et al.(2017)
	EoS					LC = 51.6			
	UCNPs					LC = 11.5			
PLA-PEG	Verteporfin	*_*	79.71 ± 5.62	0.15	N/A	LE = 100	Verteporfin release: approximately 40% with ultrasound after 24 h DOX release: 85% with ultrasound after 24 h	The solvent switch approach was used to create PLA-PEG polymersomes.	Kim et al. (2023)
	DOX					LE = 95.05 ± 0.84			
PEG-HPPH Fe and PEG-poly(linoleic acid)	HPPH Fe	_	80	N/A	N/A	N/A	DOX release: more than 60% after 20 min laser irradiation after 48 h	To synthesize PEG-HPPH Fe, PEG-HPPH was mixed with a 10-fold surplus of free iron (II) acetate in methanol and incubated for 1 hour at room temperature under an argon atmosphere.	Wang et al.(2021)
	DOX								
PEG-b-*P*-(CPTKMA-co-PEMA)	Fe_3_O_4_	*_*	176	N/A	N/A	LC = 5.6	GOx release: negligible release after 24 h CPT release: 79.2% in the presence of 1 mg/ml glucose at pH 6.5 after 72 h	The large, size-selective membrane permeability of the prepared polymersome and the high molecular weight of GOx (about 150 kDa) are responsible for the restricted GOx release.	Ke et al. (2019)
	GOx					LC = 5.2			
	CPT					N/A			

## 4. Immunotherapy-based combination therapy

### 4.1. Combination of immunotherapy

Immunotherapy, a novel approach to cancer treatment, distinguishes itself from traditional methods such as radiotherapy and chemotherapy by actively engaging and enhancing the immune system's ability to recognize and destroy cancer cells on multiple fronts. This approach aims to enhance the immune system by modulating the immune microenvironment, thereby improving immune cells' ability to identify and target tumor cells at critical points in the body (Tan et al. 2020).

However, the clinical translation of new cancer immunotherapies is slowing, as current strategies show limited effectiveness and can trigger systemic autoimmunity (June et al. 2017; Lesterhuis et al. 2011). Recently, nanomedicine has been proposed to enhance cancer immunotherapy by providing greater control over when and where immune-stimulatory cues are delivered, a capability that current approaches lack (Goldberg 2019; Irvine and Dane 2020). By leveraging nanocarrier technology, the therapeutic window of cancer immunotherapy can be expanded through precise control of antitumor immunity.

Historically, nanomedicine has sought to minimize interactions with the immune system. However, in cancer immunotherapy, the goal is the opposite: to specifically target and modulate immune cells in a controlled way. This approach requires high levels of nanoparticle accumulation in lymphoid organs. Notably, this means that the clearance of nanoparticles by phagocytic immune cells is actually beneficial rather than a hurdle to overcome. By tuning the physicochemical properties of the nanoparticle system, it is possible to control its interaction with immune cells and gain the ability to modulate the immune system (Scheerstra et al. 2022).

Due to their physical and chemical versatility, polymersomes have garnered significant attention in the field of cancer immunotherapy. Over the past few decades, research on polymersomes has led to a solid understanding of how to fine-tune their physical and chemical properties (Leong et al. 2018).

The tumor development process is complex and dynamic. Therefore, a personalized immunotherapy protocol must be devised based on the specific characteristics of the tumor and the patient's immune status. With this particular immunotherapy approach, the best possible therapeutic outcomes are aimed to be achieved, and the recovery of patients with tumors is promoted.

Immunotherapy with checkpoint inhibitors has become a standard first-line treatment for many major types of cancer. This combination therapy targets multiple factors that suppress the immune system near the tumor and initiates multiple steps in the cancer-immunity cycle. A promising class of combination immunotherapy is the combination of the anti-PD-L1 antibody atezolizumab and the antivascular endothelial growth factor antibody bevacizumab. This treatment has produced unprecedented clinical results (Lu et al. 2022).

**Figure 11.**
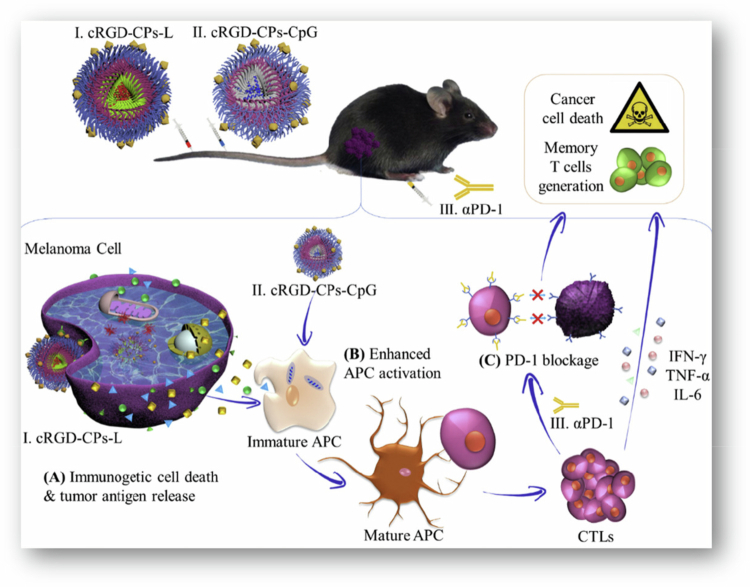
LTX-315-loaded cRGD-CPs selectively target B16F10 melanoma via i.v. injection, inducing ICD and enhancing antitumor immunity in mice when combined with CpG and anti-PD-1. Reprinted with permission from Xia et al. (2021).

In this context, a study evaluated the effectiveness of combination therapy using PD-1 antibodies and cRGD-functionalized chimeric PEG-P(TMC-DTC) polymersomes, which carry LTX-315 (an oncolytic peptide with antitumor effects) and a CpG adjuvant. The adjuvant effect of CpG ODNs enhances professional antigen-presenting cells, promoting both humoral and cellular immune responses, in C57BL/6 mice with B16F10 tumor xenografts. The oncolytic peptide LTX-315 elicits a strong immune response and causes extensive necrosis by releasing highly active tumor antigens and danger-associated molecular pattern (DAMP) molecules. cRGD supports malignant solid tumors, such as B16F10 melanoma, and tumor neovasculature, which overexpresses αvβ3 integrin. In vitro, the cRGD-chimeric polymersome of LTX-315 (cRGD-CPs-L) demonstrated potent and selective cytotoxicity against B16F10 cells (compared to L929 fibroblasts) and improved serum stability. Systemic administration of cRGD-CPs-L resulted in significant tumor accumulation of 4.8% ID/g and notable tumor growth inhibition. Moreover, the combined application of polymersomal CpG and anti-PD-1 antibody further enhanced treatment, with two out of seven animals achieving long-term immunological memory and a strong immune response. Indicators of the immunotherapeutic effect included TEM and TCM activation in the spleen, tumor infiltration of CD8 + cytotoxic T lymphocytes (CTLs), Th2 cells, and increased secretion of IL-6, IFN-*γ*, and TNF-*α* (Figure 11) (Xia et al. 2021). This biocompatible polymersome provides a promising platform for dual-surface modification with the RGD peptide and PD-1 antibody, as well as simultaneous encapsulation of peptides (LTX-315) and adjuvants (CpGs). Its chemistry offers versatile opportunities for multifunctionalization and loading of sensitive molecules such as peptides for combination immunotherapy.

### 4.2. Combination of immunotherapy and CDT, PDT, or radiotherapy

Methods to increase the effectiveness of immunotherapy and minimize side effects are being researched by identifying new targets and modalities, including combination therapies such as radiotherapy (RT), PDT or CDT (Tan et al. 2020).

Notably, PDT and CDT have been found to trigger a T cell-adaptive immune response and cause immunogenic cell death (ICD). When used alongside other treatment methods, PDT shows great potential for managing cancer (Yang et al. 2025).

Moreover, our understanding of radiotherapy (RT) as a straightforward local treatment has undergone a significant shift in recent years. It is now widely recognized that RT can trigger a systemic immune response, providing a strong basis for combining RT with immunotherapy (Zhang et al. 2022).

**Figure 12.**
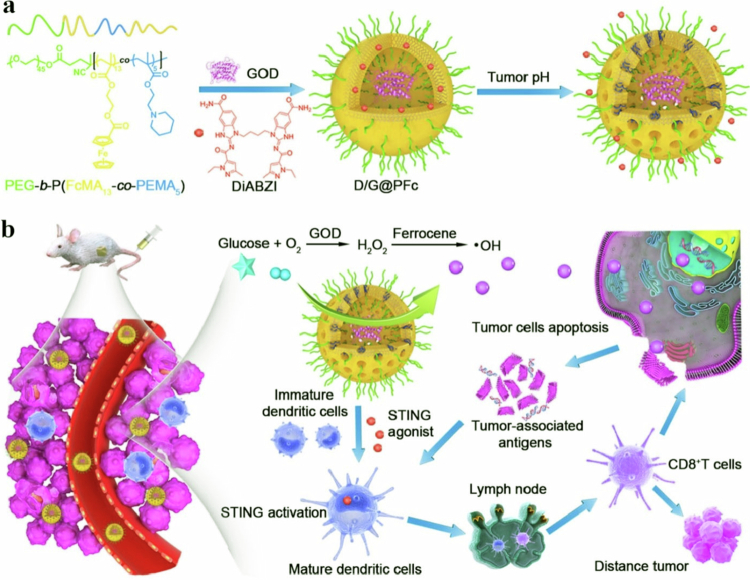
(a) Representation of the polymersome nanoreactors using self-assembly in aqueous solution at pH 7.4 to co-load GOx and DiABZI (D/G@PFc). Polymersomes exhibit membrane permeability that is responsive to tumor pH. (b) Following systemic administration, the polymersome nanoreactors produce highly toxic •OH radicals within tumor tissues through cascade catalysis involving GOx and ferrocene, enabling CDT. To reduce the immunosuppressive tumor microenvironment and enhance STING stimulation, tumor cell death facilitates the release of tumor-associated antigens and fragmented DNA. Dendritic cells can internalize the DiABZI released from the polymersomes to initiate the STING pathway, which encourages dendritic cell maturation and ultimately recruits CD8^+^ T lymphocytes. Immunotherapy and chemodynamics work together to enhance anticancer immunity. Reprinted with permission from Zhou et al. (2021).

The combined effects of immunotherapy and CDT have been evaluated in research using polymersomes as versatile carriers. In this work, GOx and symmetry-linked amidobenzimidazole (DiABZI), an agonist of stimulator of interferon genes (STING), were coloaded into ferrocene-containing polymersomes (PEG-b-P(FcMA-co-PEMA) nanoreactors) to improve STING activation and combine chemodynamics and immunotherapy in 4T1 tumor-bearing BALB/c mice. Tumor acidity-induced polymer membrane permeability facilitates the entry of tumor-derived glucose and oxygen, enabling glucose oxidase (GOx) to produce H_2_O_2_ upon intravenous injection. This is subsequently transformed into •OH by the Fenton reaction and ferrocene moieties. CDT by •OH radicals effectively induces cell death, releasing tumor-associated antigens and DNA fragments. This process preferentially involves activation of the STING pathway, strengthening the immune response and overcoming the immunosuppressive TME. DiABZI, a pH-responsive molecule, promotes antitumor immunity by activating the STING pathway. Through synergistic chemodynamic-immunotherapy, DiABZI and CDT enhance the overall anticancer response, completely eliminating primary tumors and significantly suppressing the growth of distant cancers that arise after treatment (Figure 12) (Zhou et al. 2021).

Another study created a nano-agonist for highly responsive tumor immunotherapy by encapsulating the STING agonist diABZI in P(DMA-co-MAA-g-NBS) polymersomes containing a Type I photosensitizer. The novel nanostructure facilitated H-aggregation and intersystem crossing of the Type I photosensitizer, increasing the production of ROS, including superoxide anions and singlet oxygen, by approximately threefold. To facilitate STING-based immunotherapy, PDT efficiently kills hypoxic tumor cells and triggers immunological responses, stimulating CTLs and natural killer (NK) cells. A polymersome composition, administered by intravenous injection combined with laser treatment, successfully cured mouse orthotopic mammary tumors and increased tumor-free survival by more than 50 days with rechallenge, thereby preventing tumor recurrence. These nanovesicles maximally enhance long-term survival by inducing immunogenic cell death, activating CTL and NK cell immune responses, and sustaining immunological memory, which is responsible for dampening tumor recurrence and metastasis, as demonstrated by subsequent in vivo investigations (Wang et al. 2024).

Cyclic dinucleotides, including c-diGMP, c-diAMP, and cGAMP, are a few of the STING agonists that activate the STING pathway. This results in the generation of type I IFN and other cytokines, the maturation of dendritic cells, and the cross-presentation of tumor antigens for subsequent T-cell priming. ADU-S100, a synthetic cyclic dinucleotide, has advanced into clinical trials in patients with relapsed/refractory melanoma or triple-negative breast cancer in combination with an anti-PD-1 antibody. In a previous study, ADU-S100 encapsulated in reduction-responsive biodegradable PEG-P(TMC-DTC) polymersomes significantly improved the cytosolic delivery and tumor retention of ADU-S100, enhancing STING pathway activation and antitumor immunotherapy in malignant B16F10 melanoma-bearing C57BL/6J mice. The cytosolic delivery of cancer immunotherapy was significantly improved, particularly when combined with fractionated low-dose radiation. Radiation, as a neoadjuvant anticancer therapy, works by augmenting cytoplasmic DNA sensing via the cGAS/STING pathway and by liberating tumor-associated antigens. However, fleeting STING activation by irradiation alone limits durability; low doses are usually insufficient, and high doses of radiation can cause systemic damage. These issues were resolved by combining low-dose radiation with ADU-S100-loaded polymersomes. ADU-S100-loaded polymersomes dramatically promoted CTL infiltration into tumors, decreased the number of immunosuppressive M2 macrophages, and increased STING activation to maintain antitumor immune responses for a long period of time. When combined with 5 Gy X-ray, this therapy improved survival rates, increased the percentages of mature DCs and effector T cells, and augmented IFN-*β* and TNF-*α* secretion. Some mice even showed complete tumor eradication. The prepared polymersomes also limit systemic exposure to STING agonists, which can help reduce side effects. This strategy has the potential to develop safe, effective, and long-lasting radioimmunotherapy for cancer treatment (Zheng et al. 2022).

### 4.3. Combination of immunotherapy and chemotherapy or PDT

Chemoimmunotherapy is a novel cancer treatment strategy that combines conventional chemotherapy and immunotherapy. It is designed to enhance the effectiveness of cancer treatment by directly targeting cancer cells with chemotherapy while concurrently stimulating the anticancer activity of the immune system with immunotherapeutic agents.

Research has shown that certain chemotherapy drugs can act as powerful adjuvants for boosting antitumor immunity under specific conditions, which may improve the effectiveness of immunotherapy (Pol et al. 2015). As a result, more than 200 clinical trials combining PD-1/PD-L1 blockade with chemotherapy have already been completed, and several chemoimmunotherapy combinations have recently been approved for clinical use because of their improved patient survival rates, with generally predictable safety profiles based on the known side effects of each agent (Sordo-Bahamonde et al. 2023). The combination of chemotherapeutic and immune-activating agents into a platform could provide a versatile opportunity for effective cancer treatment.

In this context, the therapeutic response of *β*-galactosidase-loaded tumor-targeting polymersomes was explicitly designed for enzyme prodrug chemoimmunotherapy targeting. The primary objective was to utilize the prodrugs *N*-(*β*-D-galactopyranosylbenzyloxy carbonyl)-DOX and *N*-(*β*-D-galactopyranosylbenzyloxy carbonyl)-NLG919 to achieve therapeutic responses with reduced toxicity in BALB/c mice with 4T1 tumors. These nanoreactors were designed by adding *β*-galactosidase to PEG-b-*P* (PEMA-co-butyl methacrylate (BMA)) polymosomes with optimized membrane cross-linking density. The resulting nanoreactor exhibited several distinctive features: enhanced stability with maximized membrane cross-linking, a minimal size of approximately 100 nm under neutral conditions for long-term circulation and active targeting of tumors, and the ability to expand to approximately 200 nm in tumor sites for prolonged retention. Additionally, the acidic tumor microenvironment can regulate membrane permeability, allowing for the selective activation of prodrugs. Upon intravenous administration, the nanoreactor accumulates at the tumor site via the EPR effect. In the tumor microenvironment, they swelled but localized to the tumor tissue for one week. Over six days, the prodrugs were administered three times, with continuous *in situ* activation by the nanoreactor at the tumor site. Activated DOX potentiates the induction of tumor-associated antigens and the elimination of cancer cells, thereby promoting the maturation of dendritic cells. Concurrently, activated NLG919 abolishes the immunosuppressive tumor environment by inhibiting indoleamine 2,3-dioxygenase 1 (IDO-1) and modulating the balance between the essential amino acid L-tryptophan and its metabolite L-kynurenine. The combination of DOX and NLG919 in chemoimmunotherapy interactions is highly therapeutically valuable for treating untreated primary tumors and distant tumors with decreased side effects (Japir et al. 2021). Another experiment reported a flexible polymersomal nanoformulation that acts as an adjuvant for *in situ* dendritic cell vaccination while facilitating tumor-associated antigen production. In this study, a PEG-poly(methyl methacrylate-co-2-amino ethyl methacrylate (thiol/amine)-poly 2-(dimethylamino)ethyl methacrylate (PEG-P(MMA-co-AEMA (SH/NH_2_)-PDMA) triblock copolymer was synthesized and self-assembled to form a chimeric cross-linked polymersome. Crosslinked polymersomes were loaded with HHPH (2-[1-hexyloxyethyl]-2-devinyl pyropheophorbide-a), a photosensitizer that helps PDT for ROS production, and low doses of DOX to induce immunogenic cell death. This combination can increase the amount of tumor-associated antigens and introduce dendritic cells, triggering a cascade of immune responses. Furthermore, when combined with tumor-associated antigens for the treatment of MC38 colorectal cancer, polymersomes containing primary and tertiary amines function as adjuvants and are capable of stimulating the recruitment of dendritic cells to create an *in situ* DC vaccination. Following a single intravenous injection of a modest dose of DOX and HPPH, *in vivo* data showed that the all-in-one polymersomal nanoformulation enhanced mature dendritic cells in tumor-draining lymph nodes and CD8^+^ T lymphocytes in tumor tissues to suppress primary and distant MC38 tumor growth in C57BL/6 mice (Yang et al. 2019).

As a result, polymersome-based drug delivery systems have shown significant promise in combination cancer therapies. By improving targeted drug delivery and immune responses, these systems can increase the effectiveness of immunotherapy, radiotherapy, chemotherapy, and PDT. The characteristics (size, PDI, zeta potential, loading efficiency or capacity, and release) of various polymersomal nanovehicles used in immunotherapy-based combination therapy are summarized in Table 3.

**Table 3.** Characterization of different polymersome-based nanocarriers used in immunotherapy-based combination therapy.

			Characterization						
Polymer	Encapsulated moiety	Targeting moiety	Size (nm)	PDI	Zeta potential (mV)	Loading efficacy (LE) or loading capacity (LC) (%)	Release (%)	Description	Reference
PEG-P(TMC-DTC)	LTX-315	cRGD	53 ± 1.8	0.19 ± 0.01	−1.2 ± 0.2	LE = 87.5	N/A	The inner shell of the cRGD-chimeric polymersome was designed with negatively charged polyaspartic acid and positively charged spermine to effectively load LTX-315 and CpG.	Xia et al.(2021)
	CpG adjuvant		42 ± 2	0.1	+1	LE ≈ 100			
PEG-b-P(FcMA-co-PEMA)	DiABZI	_	113.1	N/A	N/A	LC = 6.4	DiABZI release: more than 40% in the presence of glucose at pH 6.5 after 24 h	PPEMA segments were included in polymersome membranes to provide tumor pH-responsive membrane permeability due to the ultrasensitive pH-responsive characteristics of PPEMA polymers, which have a pKa of around 6.9.	Zhou et al. (2021)
	GOx					LC = 5.2			
P(DMA-co-MAA-g-NBS)	DiABZI	_	82	0.1	N/A	LE = 95.6	N/A	DiABZI could be efficiently added to nanovesicles by employing the nanoprecipitation technique.	Wang et al.(2024)
PEG-P(TMC-DTC)	ADU-S100	_	45.7	0.16	+0.14	LE = 86	Cy3-diAMP release: 58.9% in the presence of 10 mM GSH after 12 h	Cy3-diAMP was used as a model of cyclic dinucleotides for the study of *in vitro* release. Cyclic dinucleotide-loaded chimeric polymersomes demonstrated remarkable colloidal stability when placed in a buffer containing 10% FBS or after being stored for three weeks.	Zheng et al. (2022)
PEG-b-*P* (PEMA-co-(BMA)	*β*-galactosidase	_	117 (pH 7.4)	0.09	N/A	LC = 5.7	GOx release: more than 70% at pH 6.5 after 48 h	In order to avoid the expensive expense of *β*-galactosidase, GOx was employed to conduct several studies.	Japir et al.(2021)
			215 (pH 6.5)	0.07					
PEG-P(MMA-co-AEMA (SH/NH2)-PDMA	DOX	_	86	0.32	+3.4	LE = 82.7	DOX release: ≈ 100% in PBS after 48 h HPPH release: More than 90% in PBS after 48 h	Chimeric cross-linked polymersomes were fabricated by the solvent exchange method	Yang et al.(2019)
	HPPH					LE = 96.8			

## 5. PTT-based combination therapy

### 5.1. Combination of PTT and chemotherapy

In recent years, NIR laser-based photothermal therapy (PTT) has garnered increased interest as a promising and minimally invasive approach for eliminating cancer cells. PTT causes hyperthermia at 41–49 °C by absorbing light through photothermal agents (Hasannia et al. 2024). PTT has several advantages over conventional cancer therapy methods, including minimal invasiveness, accurate spatial‒temporal selectivity, minimal drug resistance, and outstanding specificity (Mohammadi Zonouz et al. 2024).

PTT is considered a promising approach for cancer treatment because it provides benefits such as minimal invasiveness, precise control in space and time, and fewer systemic side effects compared to traditional therapies (Xu et al. 2025). Specifically, this localized heat can directly kill tumor cells, modify the tumor microenvironment, and initiate various biological responses that enhance the overall treatment outcome. For example, near-infrared (NIR) light, typically ranging from 650 to 900 nm, is often chosen because it can penetrate deeper into tissues and has low absorption by water and hemoglobin, making it a suitable choice for targeting tumors located further inside the body. Photothermal therapy (PTT) encompasses both traditional photothermal therapy (≥45 °C) and mild photothermal therapy (Jiang et al. 2021). Conventional PTT effectively destroys tumors, but it can also damage healthy tissues because of imprecise PTA targeting and the risks associated with laser use. Low-intensity PTT can help minimize damage to healthy tissues and enhance immune responses, but it is not as effective against tumors. To achieve better results, researchers have paired PTT with other treatments, including chemotherapy, radiation therapy, photodynamic therapy, immunotherapy, and gene therapy. This approach leverages the benefits of combination therapies, addresses treatment resistance, and tackles tumor complexity (Chen et al. 2021).

The development of advanced nanomaterials has been crucial in making combination therapies a reality. Polymersomes can function as multifunctional platforms, serving as carriers for therapeutics, PTT, PDT and other agents (Mohammadi et al. 2018; Mohammadi et al., 2017). This flexibility enables the development of comprehensive treatment strategies that target multiple aspects of cancer biology.

In this context, a study investigated the possibility of employing polymersomes containing gold nanorods (AuNRs) and DOX as dual stimuli-sensitive drug delivery vehicles for cancer combination therapy. RAFT polymerization was used for the preparation of PEG-b-poly(*N*, *N*-diethylaminoethyl methacrylate) (PEG-b-PDEAEM) amphiphilic block copolymers with two various macro chain transfer compounds with PEG at 2000 and 5000 g/mol and a variable length of the PDEAEM block. Two methods were used for the preparation of polymersome-type nanometric aggregates from the resulting block copolymers. Approximately 200 nm was obtained through direct dispersion, and diameters of approximately 100 nm were achieved through solvent exchange using THF and water. The polymersomes exhibited positive zeta potential values ranging from +0.5 to +25 mV at a physiological pH of 7.4, which was dependent on the PDEAEM content. These findings are suitable for the future internalization of aggregates by cells. DLS was used to confirm the temperature responsiveness of the polymersomes. Aqueous dispersions (0.5 mg/mL, pH 7.4) displayed moderate shrinkage (5%) in size when heated from 25 to 55 °C. 37–38°C was found to be the phase transition temperature. This pH-responsive behavior is regulated by the hydrophobic–hydrophilic balance of the copolymers. Owing to the powerful ionization of the DEAEM units, there was no transition temperature between 25–55 °C at pH 5.8 for the PEG-b-PDEAEM copolymers. The results of drug delivery testing indicated that a decrease in pH from 7.4 to 5.8 and irradiation of the systems by an NIR laser at pH 7.4 increased the release rate of DOX. Enhanced drug release was achieved only by the synergy of lower pH and NIR irradiation in the case of polymersomes of lower-molecular-weight PEG (DiazDuarte-Rodriguez et al. 2019).

An advanced polymeric vesicle constructed from the triblock polymer PEO-co-poly(Cys-AuNP@FA)-co-poly(3-methoxypropylacrylamide) (PEO-co-PCF-co-PMA) with a magnetic NP encapsulated within it was used in a study that possessed the potential to deliver methotrexate with targeted delivery (with FA serving as the targeting agent), PTT (anisotropic AuNPs), and stimuli-responsive T1 imaging (as an MRI contrast agent). Upon encapsulating magnetic NPs (Gd-doped SPIONs), a polymer is formed, termed a magnetopolymersome (MPS), which is easily prepared in high yield and exhibits excellent biocompatibility and efficient NIR-responsive PTT. MPS exhibits excellent temperature and NIR T1 contrast properties in vitro and maintains good stability under both normal physiological conditions and more challenging environments, such as serum or Triton X-100. Four types of AuNP morphologies, including spherical, triangular, rod, and flower morphologies, were synthesized to investigate how the AuNP morphology affects the PTT and drug transportation properties of the resulting nanocarrier. Compared to their other counterparts, the nanoflower-conjugated MPS showed the most effective NIR-responsive action. Additionally, the increased cytocompatibility and rapid uptake of MPS by the MCF-7 cell line were confirmed by confocal microscopic imaging and the MTT assay. These characteristics make polymersomes based on nanoflowers or other anisotropic nanoparticles highly effective prospective nanocarriers for PTT, loading, delivery, and imaging (Roy et al. 2017).

Bubble-generating polymersomes, capable of producing bubbles upon exposure to low pH or hyperthermia, were created in a study to coencapsulate photosensitizing and chemotherapeutic agents for synergistic chemo-photothermal cancer therapy. DOX was encapsulated in the hydrophilic core of PCL-PEG-PCL polymersomes, and the photosensitizer ICG was loaded in the bilayer. The NH_4_HCO_3_ encoded in the bubble-producing DOX-ICG-codelivery polymersomes would degrade to form CO_2_ bubbles under acid or laser irradiation, disrupting the vesicle structure and causing rapid release of DOX. An acidic environment and NIR laser irradiation promoted DOX release from the polymersomes, as revealed from *in vitro* investigations of drug release. A cell uptake experiment demonstrated that laser-induced hyperthermia significantly increased the endocytosis of the formed polymersomes into 4T1-Luc cancer cells. According to an *in vitro* cytotoxicity experiment, bubble-generating DOX-ICG-codelivered polymersomes displayed much higher cytotoxicity under laser irradiation than did free drugs. According to an *in vivo* biodistribution experiment, the constructed polymersomes can enhance the photothermal conversion efficiency, prolong drug retention, and concentrate at the tumor site. Additionally, the synthesized polymersomes, under laser irradiation, successfully suppressed 4T1-Luc tumor growth while minimizing systemic toxicity, as shown by *in vivo* tests in BALB/c mice. Therefore, the developed bubble-generating polymersome platform has proven to be an excellent multifunctional nanovehicle for combination chemotherapy and photothermal therapy (PTT), as well as stimuli-responsive controlled drug delivery (Zhu et al. 2018).

An efficient AuNR and DOX coloaded mPEG‒PCL polymersome system was engineered in a study to enable the combination therapy of PTT and chemotherapy. The polymersomes could be disrupted by the local hyperthermic environment of the AuNRs induced by NIR laser irradiation, facilitating the release of DOX. In contrast to chemotherapy or PTT alone, the combination therapy yielded a more significant therapeutic effect, as tumors were fully suppressed without relapse when it was used for tumor ablation in C26 tumor-bearing BALB/c mice. Compared with monotherapy, combination therapy induced a greater number of cells towards the apoptotic stage, as indicated by TUNEL immunofluorescent staining of tumor tissues. Additionally, a pathological analysis of cardiac tissue revealed that AuNR- and DOX-coloaded polymersomes were less toxic systemically than free DOX (Liao et al. 2015).

In an attempt, a polymersome-based multifunctional theranostic system was designed for targeted photothermal therapy (PTT), chemotherapy, and X-ray computed tomography (CT) imaging of melanoma tumors. Amphiphilic (polyethylene glycol)-co- (poly(*γ*-benzyl-l-glutamate) was used to develop a polypeptide-based polymersome, which was then coencapsulated with lipophilic CUR and hydrophilic AuNR in the lipophilic bilayer and aqueous core of the polymersome (*P*-AuNR-CUR), respectively. Sgc8-c aptamers were then coated on the Apt-*P*-AuNR-CUR polymersome for targeted delivery. Compared to single cancer treatment, NIR-responsive polymersomes have excellent therapeutic effectiveness in chemo/photothermal therapy, as evidenced by both *in vitro* and *in vivo* studies on B16F0 cells and B16F0 tumor-bearing C57BL/6 mice. Additionally, through *in vivo* CT scan imaging six hours after intravenous injection, the diagnostic potential of the prepared NP was verified (Hasannia et al. 2024).

In another study, a multifunctional lipopolymersome was developed for melanoma-guided chemotherapy utilizing PTT and NIR imaging. The 1,2-dioleoyl-3-trimethylammonium propane (DOTAP) and PEG-PLA diblock copolymer (molar ratio of 1:1) were utilized to create a lipopolymersomal hybrid nanovesicle. The AS1411 DNA aptamer was then coated on the lipopolymersome surface (Apt-DOX-ICG-LP) for targeted delivery. The NIR-responsive release pattern of the formulated lipopolymersome was confirmed *in vitro*. The fabricated system demonstrated excellent photothermal conversion, encapsulation efficiency, and stability. According to *in vitro* experiments, the cytotoxicity of NIR-responsive lipopolymersomes was significantly higher than that of individual anticancer drugs. The hemolysis test demonstrated the blood compatibility of the prepared system for systemic administration, in which there were no strong nonspecific interactions between Apt-DOX-ICG-LP or Blank-LP and red blood cells (RBCs). Furthermore, in contrast to animals treated with either DOX-ICG-LP or Apt-DOX-ICG-LP without laser irradiation, B16F10 tumor-bearing mice treated with Apt-DOX-ICG-LP and 808 nm laser irradiation presented superior tumor regression and a positive survival profile. Six and twenty-four hours after intravenous injection, *in vivo* NIR imaging was used to assess the diagnostic potential of Apt-DOX-ICG-LP. According to these data, Apt-DOX-ICG-LP has a targeted theranostic capability that is ideal for the dual chemo-PTT and diagnostics of melanoma (Abbasi et al. 2024).

**Figure 13.**
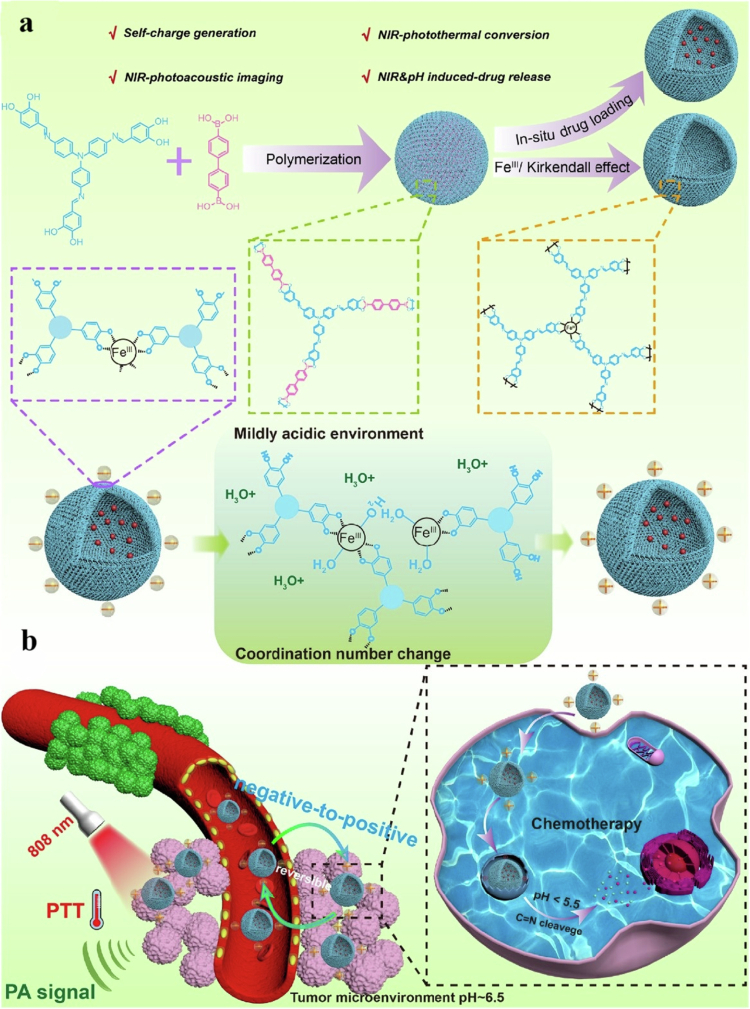
(a) Illustration of the HMPN production procedure, *in situ* drug loading for developing DOX-HMPNs, and reversible ‘negative-to-positive’ surface charge development triggered by the tumor microenvironment. b) Demonstration of photoacoustic imaging-guided, targeted, and synergistic photothermal-chemotherapy using DOX-HMPNs. Reprinted with permission from Mao et al. (2021).

Zhang et al. reported another approach for cancer therapy involving the development of a photothermal-responsive nanosized hybrid polymersome, which acts as a multifunctional therapeutic codelivery nanocarrier for efficient tumor inhibition. They synthesized porous silicon NPs conjugated with AuNRs, known as composite NPs, and encapsulated them within hybrid polymersomes. The hybrid polymersomes, composed of PEG-PLA diblock copolymers and 1,2-dioleoyl-sn-glycero-3-phosphocholine (DOPC), demonstrated high biocompatibility and low permeability. The nanovehicle demonstrated high drug-loading capabilities for hydrophobic and hydrophilic pharmaceuticals, especially for formulations of docetaxel, rapamycin, and afatinib. *In vivo* experiments with SKBR-3 HER2-positive breast cancer-bearing nude mice revealed excellent tumor inhibition of 94% and 87% at doses of 5 mg/kg and 2.5 mg/kg, respectively. The composite NPs maintained their photothermal properties, allowing drug release under NIR laser irradiation and efficiently overcoming MDR. Together, these findings show the potential of this multifunctional nanovehicle as an advanced drug delivery platform that could increase the bioavailability of previously undeliverable compounds and increase therapeutic efficacy through synergistic effects. This new platform presents promising avenues for more efficient combination therapies in cancer treatment and will present new challenges for the field of nanomedicine (Zhang et al. 2019). A study utilized a Kirkendall effect-based etching approach for the synthesis of multifunctional hollow metallosupramolecular polymer NPs (HMPNs) with photothermal conversion, photoacoustic activity, tumor microenvironment-induced self-charge generation, and pH-responsive drug release capabilities. Guest molecules, such as DOX, can be encapsulated *in situ* because of this effect. This creates vacancies in the NPs and enables the exchange of matter between the interior of the NPs and their exterior environment. The approach began with boronate polymer nanoparticles (BPNs), which were synthesized through a condensation reaction between a boronic monomer (DBB) and a catechol monomer (TAC), resulting in the development of B-*N* coordination. The boronic monomer departed, and a shell made of TAC/Fe^III^ complexes formed upon the coordination of Fe^III^ with the catechol group and the induction of the Kirkendall phenomenon. These complexes demonstrate superior photothermal and photoacoustic behaviors upon NIR light irradiation. Since the etching process facilitated efficient encapsulation with reduced premature drug leakage, DOX-loaded HMPNs could be readily prepared using DOX as a model drug. The reversible ionization of phenolic hydroxyl groups and dynamic catechol‒Fe (III) coordination endow HMPNs with the distinctive ability to self-charges. This enables the development of a surface charge in response to the mild acidity of the TME. In contrast to other charge-reversal methods based on irreversible bond cleavage or protonation, this ‘self-charge generation’ method results in a ‘negative-to-positive’ charge reversal. By reducing immunological recognition and avoiding premature clearance by the reticuloendothelial system, this charge reversal stabilizes the circulatory NPs. The DOX-HMPNs demonstrated enhanced cellular uptake, prolonged circulation, and effective tumor targeting. Furthermore, the drug was released after the degradation of the NP in acidic lysosomes. A significantly enhanced antitumor effect was achieved by combining the effects of PTT, chemotherapy, and responsive imaging in MCF-7-bearing BALB/c mice. DOX-HMPNs are potential candidates for cancer theranostic applications because of their cascade-responsive characteristics and synergistic photothermal/photoacoustic function, which can completely eliminate tumors and suppress recurrence in a single treatment cycle (Figure 13) (Mao et al. 2021).

### 5.2. Combination of PTT, PDT and chemotherapy

Photoactivation therapy, such as PDT and PTT, is emerging as a promising approach in various types of cancers. Photosensitizers and photothermal conversion agents play essential roles in the efficacy of these phototherapy techniques, particularly over the last decade. Moreover, advances in nanotechnology have significantly contributed to the progress in phototherapy for cancer treatment. PDT enhances the sensitivity of tumor cells to PTT by modifying the tumor microenvironment. In contrast, the heat from PTT can improve blood flow and oxygen delivery, consequently enhancing the therapeutic effects of PDT (Kong and Chen 2022).

In this context, the reduction-responsive copolymer PCL_7500_-ss-PEG_7500_-ss-PCL_7500_ and 1,2-distearoyl-sn-glycero-3-phosphoethanolamine-*N*-[folate(PEG)-2000] (DSPE-PEG(2000) Folate) were used in a study to develop oxygen- and bubble-producing polymersomes. The hydrophobic photosensitizers ICG and MnO_2_ were loaded into the hydrophobic layer of the membrane, and an NH_4_HCO_3_ solution was loaded into the hydrophilic inner cavity. These polymersomes exhibit multiresponsive behavior to temperature, reduction, and pH, making it possible to treat cancer with a combined therapy that is highly effective and utilizes PDT and PTT. The breaking of the copolymer's disulfide bonds in the tumor microenvironment's reducing environment, along with the resulting CO_2_ bubbles from laser irradiation or NH_4_HCO_3_ decomposition in an acidic environment, destabilizes the vesicle structure, leading to drug release. Moreover, the vigorous reaction of MnO_2_ with endogenous H_2_O_2_ can produce oxygen, thereby reversing tumor hypoxia. According to *in vitro* investigations, the polymersomes demonstrated excellent photothermal efficacy, facilitated the generation of ROS and oxygen, and efficiently killed tumor cells. According to *in vivo* experiments using BALB/c mice with CT26 tumors, oxygen- and bubble-releasing polymersomes successfully circumvent the hypoxic microenvironment in tumors, substantially suppress tumor growth, and maintain high biocompatibility (Li et al. 2021).

In another investigation, PCL-PEG-PCL and DSPE-PEG(2000) Folate were used to prepare laser-activated and targeted polymersomes that could simultaneously encapsulate the phase change agents perfluorohexane and ICG. As shown by ICG fluorescence imaging, the prepared NPs were nanosized and efficiently accumulated inside tumors. The hyperthermia caused by the ICG-loaded polymersomes could reach the boiling point of perfluorohexane (56 °C) and the melting point of the copolymers upon laser irradiation, converting perfluorohexane from a liquid to a gas. The developed polymersomes also experienced balloon-like enlargement without structural damage. The expanded polymersomes merged with neighboring polymersomes to generate significantly larger microparticles. This ‘nano-to-micro’ transformation and fusion mechanism was established to be temperature responsive, and the resulting polymersomes displayed significantly enhanced ultrasonic signals. Moreover, BEL-7402 tumor-bearing BALB/c nude mice treated with the formed NPs via ICG-mediated PTT/PDT combination therapy showed remarkable antitumor activity. Together, the innovative polymersomes produced outstanding dual imaging-guided tumor phototherapy using ultrasound and fluorescence, offering a promising option for theranostic applications (Huang et al. 2024). By using versatile stereoregular amphiphilic mPEG-block-poly(*β*-aminoacrylate)-block-mPEG (mPEG-b-PBAC-b-mPEG) copolymers, a new model of polymosomes with ROS singlet oxygen (^1^O_2_)-responsive membranes were developed. The anticancer medication DOX and the hydrophobic NIR photosensitizer IR-780 could be easily coencapsulated in these polymersomes. After intraperitoneal injection of the IR-780/DOX-coloaded polymersomes, irradiation of the tumors with NIR light promoted the tumor-specific release of DOX by disassembling the polymersome nanostructure through O_2_-mediated membrane photocleavage. Furthermore, in addition to producing ^1^O_2_, the IR-780 dye can transform NIR light energy into heat, enabling the realization of PTT and PDT. Combining phototherapy with NIR light-triggered chemotherapy using IR-780 and DOX coencapsulated polymersomes has demonstrated cytotoxicity against 4T1 cancer cells and tumor inhibition in 4T1 tumor-bearing BALB/c mice (Saravanakumar et al. 2020).

In another study, a Cis-aconitine (CA)-functionalized PEG‒poly(nitrate trimethylene carbonate) copolymer (mPEG‒PNTC‒CA) was used to prepare NO-containing polymersomes. A significant number of NO donors are naturally formed on the polymer chains to maintain stability.

These polymersomes can simultaneously be encapsulated with DOX in the lumen and the photosensitizer IR780 iodide on the membrane layer. The reducing circumstances could initiate NO release, which is further accelerated by remote NIR irradiation due to the elevated local temperature. High concentrations of immediate NO release dramatically reduce *P*-gp expression, making chemotherapy more sensitive and overcoming MDR in tumors, thereby enhancing antitumor efficacy. In addition, the cleavage of acid-labile CA amide at the endo/lysosomal pH and the hydrophilicity of the membrane layer following NO release significantly increase DOX release under intracellular conditions. The *in vivo* findings demonstrated that adriamycin-resistant MCF-7 tumor-bearing nude mice responded well to a single intravenous injection of a polymersome formulation combined with NIR irradiation. This combination of treatments results in the total elimination of tumors and minimal adverse effects (Liu et al. 2021).

### 5.3. Multi-combination therapy: PTT, PDT, chemotherapy, and antiangiogenic therapy

For photochemo-antiangiogenic therapy against cancer, folate-coated PCL-PEG-PCL polymosomes with stimuli-responsive properties were prepared and therapeutically assessed using optical coherence tomography angiography (OCTA) dual imaging and NIR fluorescence. DSPE-PEG-Folate was added to the drug-loaded polymersomes to enable active tumor targeting and folate receptor-mediated endocytosis. The folate-targeted polymersomes were coloaded with ICG and sorafenib (SOR) in their hydrophobic membrane and ammonium bicarbonate and mitoxantrone (MIT) into their hydrophilic core. The efficient accumulation and retention of the prepared NPs in the tumors were determined via NIR fluorescence imaging. The ICG generated hyperthermia and ROS for effective PTT and PDT after exposure to an 808 nm laser. Furthermore, drug release was enhanced by the breakdown of ammonium bicarbonate in response to an acidic tumor environment and ICG-mediated hyperthermia. Through FA targeting and EPR, the formulated polymersomes efficiently accumulated and entered tumors following intravenous administration. While the released SOR inhibited tumor vascularization, the released MIT entered the nucleus to prevent DNA synthesis. Notably, manufactured polymersomes clearly reduced the vascular area density when OCTA imaging was used to evaluate tumor blood flow during combination therapy. Additionally, 40% of the 4T1 tumor-bearing mice were fully cured with no recurrence, owing to synergistic photochemoantiangiogenic therapy with formulated polymersomes. The multifunctional polymersomes offer a promising synergistic approach to cancer therapy (Wu et al. 2022).

In another study, a photoallosteric polymersome formed via self-assembly of the amphiphilic derivative of poly(L-cysteine), and the photosensitizer IR-780 was loaded into it. When exposed to NIR light irradiation, this polymersome quickly changes its conformation from a *β*-sheet to an *α*-helix. The formulated polymersome was coloaded with the hydrophobic kinase inhibitor SOR and the hydrophilic cytolytic peptide Mel. In addition to enhancing NP cell penetration and lysosomal escape, photoinduced allosteric behavior restored the vesicular membrane structure, which, in turn, triggered the release of SOR and Mel. Combining phototherapy with the on-demand release of various therapies induced immune-triggered cell death and apoptosis in tumor cells, altered the immune microenvironment, and produced effective anticancer effects in 4T1 tumor-bearing BALB/c mice, thereby preventing tumor metastasis and recurrence (Zheng et al. 2023).

The characteristics (size, PDI, zeta potential, loading efficacy or capacity, and release) of different polymersomal nanocarriers used in PTT-based combination therapy are represented in Table 4.

**Table 4.** Characterization of different polymersome-based nanocarriers used in PTT-based combination therapy.

			Characterization						
Polymer	Encapsulated moiety	Targeting moiety	Size (nm)	PDI	Zeta potential (mV)	Loading efficacy (LE) or loading capacity (LC) (%)	Release (%)	Description	Reference
PEG-b-PDEAEM	AuNRs	_	131 to 281 (differed polymersomes loaded with AuNR-761 and AuNR-846)	N/A	N/A	LE = 33.8 to 44.7	DOX release in PEG_51_-b-PDEAEM_47_: approximately 43% at pH 5.8 after NIR irradiation within 24 h	Two distinct techniques were employed in this work to prepare the copolymer aggregates: solvent displacement or nanoprecipitation using THF/water and direct dispersion in distilled water.	DiazDuarte-Rodriguez et al.(2019)
	DOX					LE = 44.3 to 47.4			
PEO-co-PCF-co-PMA	AuNFs	Folate	101.0 ± 1.4	0.11 ± 0.2	N/A	LE = 87.0 ± 1.1	Methotrexate release: Approximately 95% in PBS medium at 42 °C after 5 min and 98% in PBS medium with 2 W.cm^−^^2^ power density source of NIR after 5 min	The PEO-co-PCF-co-PMA polymer vesicle was first synthesized by film rehydration, and MPS was subsequently prepared using the soaking method.	Roy et al.(2017)
	Methotrexate					LE = 95			
	Gd-doped SPIONs					N/A			
PCL-PEG-PCL	ICG	_	208.1 ± 3.5	0.278 ± 0.013	−16.4 ± 0.5	LE = 82.32 ± 1.37	DOX release: Approximately 95% at pH 5.5 with laser irradiation after 21 days	Using the thin film re-hydration technique, ICG was loaded within the bilayer of polymersomes. A transmembrane gradient loading technique with ammonium bicarbonate was used to load DOX into the hydrophilic lumen.	Zhu et al. (2018)
	DOX					LE = 14.55 ± 1.61			
mPEG-PCL	AuNRs	*_*	≈208	N/A	−5.1	N/A	DOX release: almost 70% at pH 5.0 with laser irradiation after 30 h	The double emulsion approach was used to prepare DOX and AuNRs co-loaded polymersomes.	Liao et al.(2015)
	DOX					LE = 84.3			
PEG-PLG	AuNRs	Sgc8-c aptamer	206 ± 52	0.406 ± 0.056	−4.56 ± 2.9	N/A	CUR release: about 24% at pH 5.4 after laser irradiation after 240 h	Polymersomes were prepared using the double emulsion technique. Elevating the temperature is known to damage the integrity of the self-assembled structure and accelerate CUR release by breaking hydrogen bonds and decreasing the solubility of hydrophilic segments.	Hasannia et al.(2024)
	CUR					LE = 65 ± 1.5			
PEG-PLA:DOTAP	ICG	AS1411 aptamer	74 ± 5	0.4 ± 0.4	12	LE = 99.3 ± 0.6	DOX release: approximately 60% at pH 5.5 after 808 nm laser irradiation after 144 h	The developed system was fabricated via film rehydration method for DOX and ICG loading To enhance ICG loading and decrease its dispersion through electrostatic interactions, DOTAP was used for lipopolymersome production in this work because of the negative charge of ICG	Abbasi et al. (2024)
	DOX					LE = 81.1 ± 4.7			
PEG-PLA:DOPC	Docetaxel	_	286 ± 79	0.16 (empty nanovehicle)	N/A	LC ≈ 20 (maximum amount for each drug)	Rapamycin release: Over 15% after laser irradiation compared to 5% without laser irradiation within 30 min	The composite NPs are encapsulated within the hybrid polymersomes using a microfluidic double-emulsion process, which provides exact control over the size and uniformity of polymersomes.	Zhang et al.(2019)
	Rapamycin								
	Afatinib								
Fe^III^/TAC coordination cross-linked polymer	DOX	*_*	≈200	N/A	≈−6.45 to 7.16 (from pH 7.4 to pH 6.5)	LC = 18.6	DOX release: About 76% at pH 5.5 after laser irradiation within 24 h	NPs were prepared based on the Kirkendall effect. The formulated NPs exhibit a strong negative charge at physiological pH, which transitions to a neutral and then positive charge in response to changes in pH due to the dynamic nature of the TAC/Fe^III^ complex. There is no noticeable decrease in the NP diameter throughout the self-charge generation process.	Mao et al. (2021)
	Fe^III^					N/A			
PCL_7500_-ss-PEG_7500_-ss-PCL_7500_	ICG	Folate	170.7 ± N/A	0.112 ± 0.011	−8.63 ± 0.66	LE = 75.17 ± 1.11	N/A	Oxygen- and bubble-generating polymersomes were prepared using a thin film rehydration method. The average size and polydispersity of the polymersomes increased under various conditions, suggesting that the laser, pH, or reduction caused the polymersomes to undergo structural breakdown.	Li et al. (2021)
	MnO_2_					N/A			
PCL-PEG-PCL	ICG	Folate	215.8	0.177	−27.6	LE = 93.20%	N/A	The formulated polymersomes were fabricated using a two-step emulsion method. The polymersomes loaded with perfluorohexane remained stable in an *in vivo* environment and did not expand at 37 °C.	Huang et al. (2024)
	perfluorohexane					N/A			
mPEG-b-PBAC-b-mPEG	IR-780	_	259 ± 4	N/A	N/A	LE = 45.50 ± 6.46	DOX release: almost 63% at pH 7.4 after laser irradiation with a power density of 2 W. cm^−2^ within 24 h	The formulated polymersomes were prepared via the dialysis method. The *π*-*π* interactions between the IR-780, DOX, and PBAC membrane structure of polymersomes may enhance the overall drug-loading contents.	Saravanakumar et al. (2020)
	DOX					LE = 53.92 ± 7.42			
mPEG-PNTC-CA	IR-780	_	118	N/A	N/A	N/A	NO release: 10.6% at pH 5.5 in the presence of 10 mM GSH after 48 h DOX release: 87% at pH 5.5 in the presence of 10 mM GSH after 24 h	The solvent exchange approach was used to prepare the polymersomes. The negatively charged CA moieties present within the hydrophilic interior of polymersomes promote the encapsulation of DOX through electrostatic interactions.	
	DOX					LE = over 80			
PCL-PEG-PCL	ICG	Folate	206 ± 11.42	0.28 ± 0.05	≈−17		MIT release: Completely released at pH 5.5 after laser irradiation within 168 h SOR release: 88.40% at pH 5.5 after laser irradiation within 168 h	Thin-film hydration and concentration gradient diffusion were employed to prepare the formulated nanoparticles.	Wu et al. (2022)
	MIT								
	SOR								
poly(L-cysteine) derivative	IR-780	*_*	168.5 (size of IR-780 loaded polymersome)	N/A	N/A	N/A	Mel release: about 85% at pH 7.4 after 808 nm laser irradiation within 12 h SOR release: about 85% at pH 7.4 after 808 nm laser irradiation within 12 h	The developed polymersomes were formed using a dialysis technique. The capacity of nanocarriers to penetrate cells may be enhanced by the photoinduced conversion of *β*-sheet to *α*-helix since *α*-helical polypeptides have demonstrated vigorous membrane activities associated with their helical shapes.	Zheng et al. (2023)
	Mel					LE ≈ 43			
	SOR					LE ≈ 52			

## 6. Gene-based combination therapy

Gene therapy is the most promising type of treatment because, by specifically targeting disease-inducing genes, patients can be precisely and uniquely treated for several harmful conditions. Addressing specific tissue types in patients with targeted nucleic acid molecules enables the alteration of patients' gene expression, either by inhibition, enhancement, or restoration. Molecules such as small interfering RNAs (siRNAs), microRNAs (miRNAs), and inhibitory antisense oligonucleotides (ASOs) are used extensively to downregulate gene expression. In contrast, plasmid DNA, messenger RNA (mRNA), small activating RNA (saRNA), splicing-modulatory antisense oligonucleotides (ASOs), and CRISPR/Cas platforms are typically used to enhance or correct gene expression. Several therapeutic strategies are being investigated to target specific diseases. RNA interference (RNAi) is a physiological defense mechanism that protects against the intrusion of foreign genes. RNAi technology, including siRNA and miRNA, can effectively suppress the expression of target genes in a sequence-specific manner by inducing the degradation of the targeted mRNA (in the case of siRNA and miRNA) or inhibiting mRNA translation (in the case of miRNA) (Hu et al. 2020).

Therapy, which involves the combination of siRNA and chemotherapy, offers additional advantages by simultaneously modulating several mechanisms and regulatory proteins implicated in the growth, development, metastatic spread, and therapeutic resistance of tumor cells, resulting in an overall enhanced therapeutic effect (Kumar et al. 2022).

In this context, the synthesis and characterization of novel diblock co-polypeptides, poly (aspartic acid (2-(diisopropylamino)ethyl) -b-PLys) (PAsp(DIP)-b-PLys) was reported as a pH-responsive carrier for the co-delivery of siRNA and the chemotherapeutic agent, DOX, in an investigation. The synthesized PAsp(DIP)-b-PLys could self-assemble into stable carriers at neutral pH, while disassembly occurred under acidic conditions, mimicking the endosomal and lysosomal environment. This imparts pH sensitivity, allowing the controlled release of entrapped molecules and thereby enhancing their therapeutic activity. The formed copolypeptides exhibited specific proton-buffering properties that are desirable for promoting drug release in the acidic tumor microenvironment. *In vitro* experiments with human hepatic carcinoma HepG2 cells demonstrated effective cellular uptake and intracellular release of both DOX and siRNA, indicating the potential of this codelivery system for overcoming drug resistance and enhancing the effectiveness of cancer treatment. The results indicate that such pH-responsive polymersomes can be utilized as an integral platform for codelivering chemotherapy and gene therapy and hence have excellent promise for increasing the efficacy of cancer treatments with fewer side effects (Li et al. 2015).

In another study, biodegradable polymersomes were prepared for the codelivery of siRNAs targeting the antiapoptotic gene Bcl‒xL and the chemotherapeutic drug DOX. In this system, mPEG-b-PLA copolymers were utilized for the preparation of Bcl-xL siRNA and DOX-encapsulating polymosomes. The findings showed that coencapsulation resulted in significantly more potent inhibition of cancer cell proliferation in human gastric cancer cell lines (MKN-45 and MKN-28) compared to single delivery. This further demonstrated the efficiency of coencapsulated polymosomes in triggering apoptosis through the downregulation of Bcl-xL and the upregulation of the proapoptotic gene Bax, which was achieved by the simultaneous delivery of both compounds. Furthermore, the hydrolytic degradation of mPEG-b-PLA copolymers was also examined, and the copolymers were found to be stable and sustained in acidic tumor microenvironments. Release kinetics indicate that DOX is released more rapidly than is siRNA, which is beneficial for achieving an instantaneous therapeutic response. These findings suggest that designed polymersomes may be a potential way to improve cancer therapy efficacy by codelivering chemical and genetic therapeutics, potentially increasing patient outcomes (Kim et al. 2013).

Another investigation was conducted to design an innovative Angiopep-2 decorated PEG-block-poly-(2,2,3,3-tetrafluoropropyl methacrylate) (Ang-PEG-b-PFPMA) polymer delivery system that achieves simultaneous codelivery of temozolomide (TMZ) and siRNA against retinoblastoma binding protein 4 (RBBP4) for enhancing therapeutic efficacy in glioblastoma treatment. This novel polymersomal formulation significantly improved the pharmacokinetic profiles of both TMZ and siRNA, prolonging their circulation time in blood while facilitating their effective crossing of the BBB and ensuring targeted accumulation in glioblastoma tumors. The aforementioned research revealed how synergistic antitumor effects are achieved through cotreatment with siRBBP4 and TMZ. Targeted delivery of siRBBP4, by regulating the expression level of the DNA repair enzyme O6-methylguanine-DNA methyltransferase, successfully restored the TMZ sensitivity of glioblastoma cells, which was otherwise lost due to inherent mechanisms of TMZ resistance in glioma. Researchers have demonstrated that this combination treatment suppressed tumor progression and substantially increased the survival of orthotopic U87MG-luc glioblastoma cells in mouse models, potentially with therapeutic implications for the management of this disease (Zheng et al. 2022).

Another investigation focused on the design of a fine-tuned polymer nanoassembly for the simultaneous delivery of chemotherapeutic drugs and siRNA. They synthesized an amphiphilic triblock copolymer comprising mPEG-PLys-PDPA with a hydrophilic diblock and a hydrophobic block. The copolymer is capable of forming diverse nanoscale assemblies, including micelles, polymersomes, and microparticles, based on the PDPA block length. This study highlights the importance of simultaneously delivering DOX and siArf6 siRNA into PC-3 prostate cancer cells. The polymersomes efficiently triggered lysosomal pH release of the cargos, resulting in a synergistic anticancer cell-killing effect. This study highlights the potential of formulated polymersomes as effective nanomedicines for anticancer therapy, demonstrating the advantages of polymeric carriers in enhancing drug delivery efficacy and specificity. The experimental results indicated that the prepared copolymers are capable of self-assembling into specific structures, enabling them to encapsulate and release DOX and siRNA. Therefore, optimizing the block length in the copolymer is crucial for maximizing this delivery system for enhanced anticancer therapy via concomitant interference with multiple signaling pathways (Yuan et al. 2023). Bevacizumab, a humanized monoclonal antibody that binds to all circulating, soluble VEGF-A isoforms, was the first antiangiogenic treatment to be made available. Bevacizumab suppresses the activation of VEGF signaling pathways that promote neovascularization by binding to VEGF-A and blocking its interaction with VEGFR (Garcia et al. 2020). In a study, MDA-MB-231-Luc-bearing NOD-SCID mice were used to test the effectiveness of bevacizumab and neuropilin-1 (NRP-1)-targeted chimeric polymersomes containing nucleus accumbens-associated protein-1 (NAC-1) siRNA (tLyP-1-Ps-siNAC-1) in combination therapy. In this context, the tLyP-1-PEG-P(TMC-co-DTC) copolymer was used for polymersome formation. Increased migration, proliferation, and tumor recurrence of cancer cells are associated with elevated NAC-1 expression. By upregulating hypoxia-inducible factor-1α (HIF-1α), which increases glycolysis in cancer cells, NAC-1 creates a hypoxic tumor environment. Lowering NAC-1 expression can increase the efficacy of antiangiogenic therapy, regulate stem cell differentiation, and mitigate the hypoxic environment. NAC-1 mRNA and the associated oncoprotein were significantly silenced in MDA-MB-231 cells as a result of the effective cytoplasmic transport of siNAC-1. As revealed by transwell invasion and wound healing assays, tLyP-1-Ps-siNAC-1 significantly reduced the migration and invasion of MDA-MB-231 cells. Interestingly, tLyP-1-Ps-siNAC-1 greatly enhanced the therapeutic index of bevacizumab for the treatment of metastatic triple-negative breast cancer, reducing the incidence of lung metastases and increasing the survival rate (Wang et al. 2020).

Table 5 presents the characteristics (size, PDI, zeta potential, loading efficacy or capacity, and release) of various polymersomal nanocarriers employed in oligonucleotide-based combination therapy.

**Table 5.** Characterization of different polymersome-based nanocarriers used in oligonucleotide-based combination therapy.

			Characterization						
Polymer	Encapsulated moiety	Targeting moiety	Size (nm)	PDI	Zeta potential (mV)	Loading efficacy (LE) or loading capacity (LC) (%)	Release (%)	Description	Reference
PAsp(DIP)-b-PLys	siRNA	_	203	N/A	31.9	N/A	DOX release: more than 80% at pH 5.0 after 24 h.	The PLAsp(DIP) block with the pH-sensitive ternary amine group DIP demonstrated proton-buffering capabilities in a pH range between 5.5 and 7.4, which may be utilized to intelligently control the release of anticancer medicines. Additionally, the PLys block with a primary amine group formed a complex with siRNA.	Li et al.(2015)
	DOX					LC = 6.4			
mPEG-b-PLA	Bcl-xL siRNA	_	168.9 ± 8.3	N/A	N/A	LE: 54.7	Bcl-xL siRNA release: approximately 68% at pH 5.5 after 30 h DOX release: approximately 76% at pH 5.5 after 30 h	The thin film hydration/extrusion technique was used to generate polymersomes.	Kim et al.(2013)
	DOX					LE: 32.2			
Ang-PEG-b-PFPMA	RBBP4 siRNA	Angiopep-2	≈ 90	N/A	N/A	N/A	N/A	The solvent exchange approach was used for developing the formulated polymersomes. It is anticipated that the fluorinated hydrophobic middle layer will both encapsulate TMZ and stabilize the self-assembled polymeric NPs.	Zheng et al. (2022)
	TMZ					LE = 28.4			
mPEG-PLys-PDPA	Arf6 siRNA	_	136.7 ± 6.5	0.20	+ 15.8 ± 1.4	N/A	siRNA release: 91.7 ± 5.2% in the presence of heparin at pH 5.0 in 12 h DOX release: 90.7 ± 8.0%, at pH 5.0 in 12 h	The double emulsion approach was used to create DOX-loaded polymersomes under sonication, and the electrostatic interaction between negatively charged siRNA and cationic DOX- In a pH 5.0 + heparin solution, siRNA was released quickly due to competitive binding to the cationic copolymer of negatively charged heparin.	Yuan et al. (2023)
	DOX					LE = 80.5			
tLyP-1-PEG-P(TMC-co-DTC)	NAC-1 siRNA	NRP-1	48.5	0.13	−9.2	LE = 86	fast release under 10 mM GSH (analyzed by gel retardation assay)	The low PDI and reduced size of tLyP-1-Ps-siNAC-1 compared to the blank tLyP-1-Ps suggest a uniform distribution of siNAC-1.	Wang et al.(2020)

## 7. Conclusion and future perspective

Simultaneous treatment with chemotherapeutic drugs can improve cancer therapy, but many factors greatly limit its success. These include poor penetration of anticancer drugs into tumor tissues, which requires high doses, causes rapid clearance from the body, and is often worsened by poor solubility and low bioavailability. Therefore, there is a critical need to develop an optimal drug delivery system that not only increases the effectiveness of these drugs but also reduces their harmful side effects.

The objective of using polymersome-based anticancer treatments is to improve drug effectiveness, reduce toxicity, increase targeting ability, and address several limitations. To achieve this goal, various strategies have been extensively studied, including modifying polymersome features such as polymer composition, charge, surface functionalization, adding target-specific ligands, and optimizing the formulation.

The therapeutic use of polymeric nanocarriers has demonstrated significant advantages, particularly due to their ability to target regions with increased vascular permeability and minimize side effects associated with free drugs. Combining more than one therapeutic agent into a single carrier creates new treatment opportunities but also introduces several challenges. To find an ideal drug combination, extensive biological testing is needed, which requires a deep understanding of the underlying molecular mechanisms. Additionally, identifying the best mass ratio of each drug in a codelivery system for simultaneous delivery is essential, requiring systematic research on how drug ratios affect biological activity in co-delivery systems.

Assessing the release kinetics of each component in simultaneous multidrug delivery is essential for determining the optimal ratios, as the presence of one drug can affect the release behavior of another therapeutic agent and thus its effectiveness. Additionally, the clinical development of such codelivery platforms is challenging, primarily owing to the high costs associated with creating these complex formulations. However, polymersomal codelivery therapeutics are likely to be welcomed by pharmaceutical companies as new opportunities to increase patient benefits beyond existing blockbuster drugs. Polymersomal codelivery system technology has become a promising method for more effective cancer treatment, and we can expect the development of many more polymersome-based clinical codelivery products in the near future.

GMP-grade polymersome production, which involves the creation of nanoscale vesicles through the self-assembly of amphiphilic block copolymers, requires careful control of multiple parameters to ensure reproducibility, stability, and safety for pharmaceutical use. This process involves selecting suitable materials, optimizing the self-assembly method, and implementing rigorous quality control protocols. Essential factors in GMP-compliant polymersome manufacturing include the following:

(1) Materials selection: Amphiphilic block copolymers: The choice of copolymer is of foremost importance, as it dictates the polymersome's structure and stability, as well as its drug loading capability. PEG-b-PLA, PEG-b-PCL, and other materials with tunable properties are utilized regularly.

(2) Solvents: Solvents (and nonsolvents) used for the self-assembly process need to be chosen because of their biocompatibility, pharmaceutical acceptability, and capacity for removal during processing.

(3) Self-assembly process: (i) Nanoprecipitation/solvent shift: This is the most frequent method, where a solution of the copolymer in a good solvent is blended with a nonsolvent, and the copolymer self-assembles to create polymersomes. (ii) Microfluidics: Microfluidic devices provide precise control over the conditions of mixing, leading to more homogeneous polymersomes with controlled size and distribution. (iii) Thin-film hydration and other methods: Methods such as thin-film hydration, direct hydration, and electroporation could also be scaled up for GMP manufacturing.

(4) Process optimization and control: Process parameters such as temperature, pH, flow rates (in the case of microfluidics), and solvent-to-nonsolvent ratios have to be well controlled to obtain batch-to-batch consistency.

(5) Sterilization: Polymersomes for pharmaceutical applications must be sterilized (e.g. by filtration or autoclaving).

(6) Quality control: (i) Characterization employs rigorous techniques such as dynamic light scattering, electron microscopy, and high-performance liquid chromatography (HPLC) to verify the size, shape, and drug loading of polymersomes. (ii) Stability studies are essential for assessing the shelf-life and stability of polymersomes under various conditions.

(7) Scale-up: (i) Moving from the laboratory to manufacturing presents a significant challenge in scaling the laboratory process to large-scale GMP production while maintaining the desired properties. (ii) Process validation: It is essential to fully validate the process to ensure that manufacturing consistently produces polymersomes that meet the target specifications.

By rigorously addressing these areas, robust and scalable GMP processes for polymerosme production can be established, paving the way for their successful translation to the clinic.

AI-driven polymer design is transforming the creation of polymersomes, making the process faster, more efficient, and cost-effective for various applications, especially in drug delivery. By using machine learning, researchers can predict polymer properties, improve synthesis methods, and discover new monomers, which significantly reduces costs and development time. AI models can be used to predict properties such as the glass transition temperature (Tg), thermal conductivity, and tensile strength of a polymer's molecular structure. This enables scientists to pre-assess many potential designs and select candidates that meet specific application requirements before synthesis. For example, AI can estimate how polymersomes perform in drug delivery, enhancing drug encapsulation, protection, and targeted release. It can also identify optimal synthesis pathways by analyzing factors such as monomer choices, reaction conditions, and polymer architecture, streamlining manufacturing and improving yield and quality. Additionally, machine learning helps predict the properties of polymer blends, supporting the development of greener, more sustainable materials.

AI enables solving the ‘inverse design’ challenge, where users specify a polymersome's desired property, and the system calculates the best polymer structure to achieve it. This ability is beneficial in applications that require precise control of polymersome behavior, such as targeted drug delivery. AI also helps overcome obstacles in polymer design, including limited data and system complexity. By utilizing advanced machine learning methods, such as generative adversarial networks and variational autoencoders, researchers can explore a wide range of polymer possibilities and discover new materials with targeted properties.

Interpretable AI models and shared data platforms are also being developed to increase the credibility and applicability of AI-assisted polymer design.

Polymersomes offer several benefits over their lipid counterparts, causing many to believe that they will become next-generation drug delivery systems. However, lipid nanoparticles, comprising liposomes and solid lipid nanoparticles, still dominate the clinical landscape, while polymersomes remain absent from clinical trials (Matoori and Leroux 2020).

Several factors hinder the clinical translation of polymersomes. First, only a limited number of polymers, such as PEG and PLGA, have received US Food and Drug Administration (FDA) approval, while most lipids are already approved for clinical use because of their inherent biocompatibility. Additionally, methods for large-scale and reproducible production of polymersomes are limited, whereas this process is well established for LNPs. Moreover, the chemical versatility of polymersomes expands design options, but it also makes comparing studies involving structurally and chemically different systems more difficult. As a result, the clinical translation of polymersomes remains challenging, despite their potential as versatile and customizable platforms, particularly in cancer treatment.

Overall, this review highlights the potential of polymersomes as platforms for combination therapy for cancer. Polymersomes have several benefits over traditional systems and can become a unified multivalent platform, which is particularly well suited for, but not limited to, cancer combination therapy.

## Supplementary Material

Supplementary material Permissions.docx

## Data Availability

No data and materials were used for the research described in the article.
