# Supplementary figures and images for: Polymersome-based nanomedicine for combined cancer therapy
Source: Drug Deliv. 2025 Dec 22;33(1):2577137. doi: 10.1080/10717544.2025.2577137 (PMC12724104; doi:10.1080/10717544.2025.2577137)

Figure 11


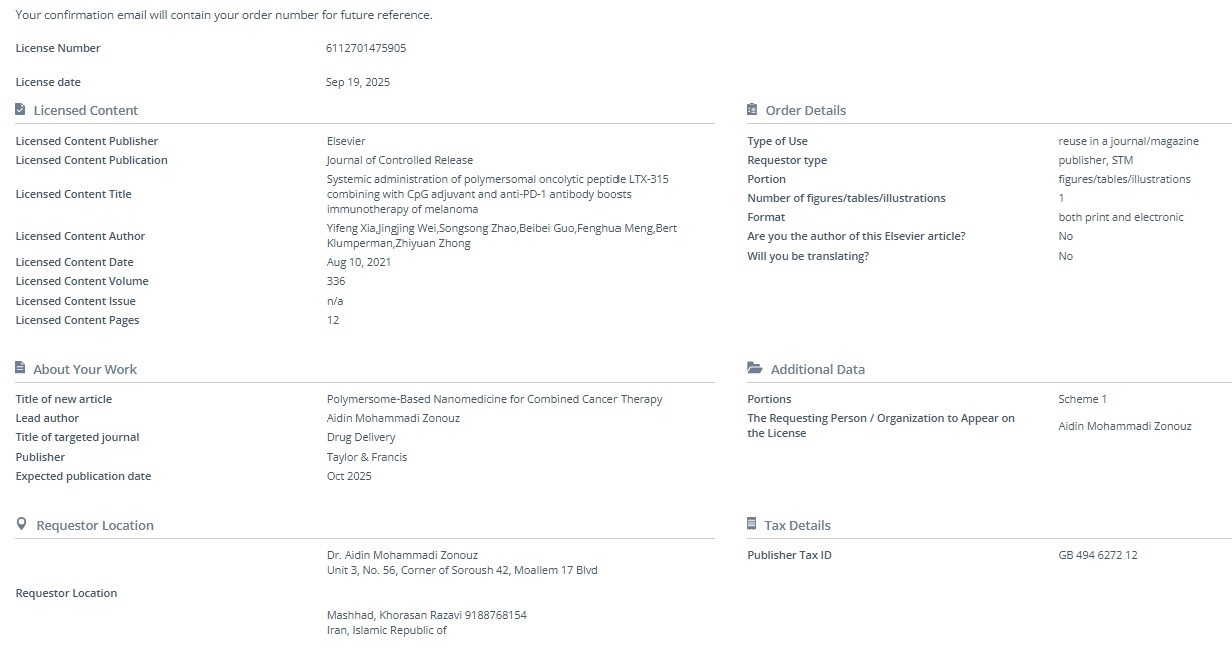


Figure 12


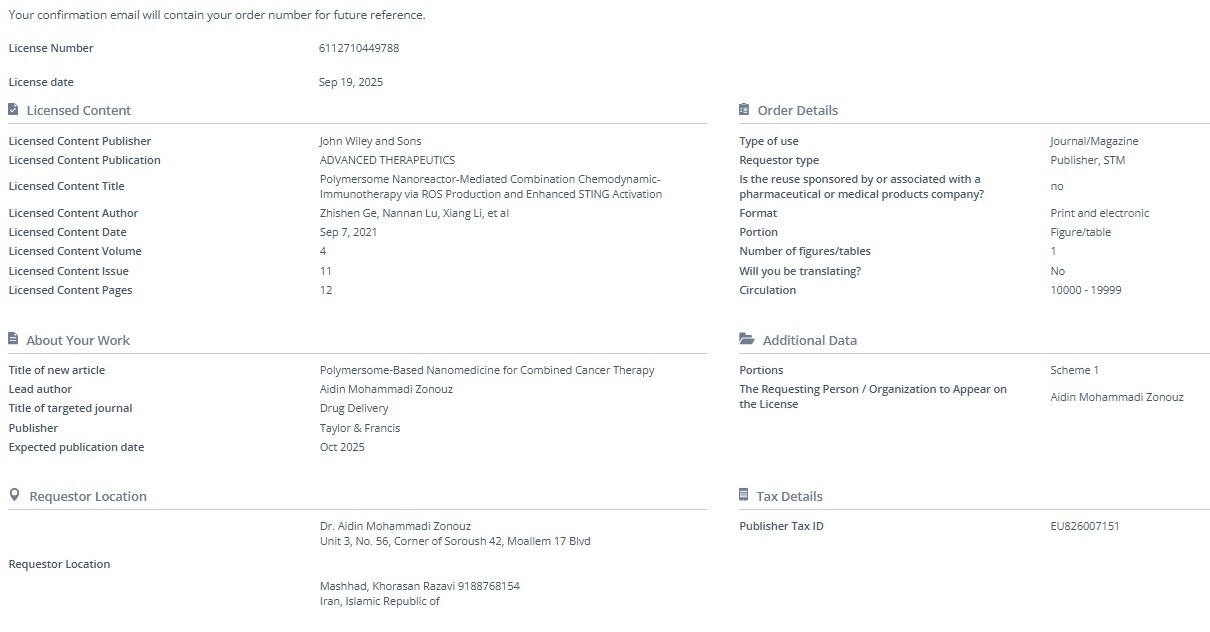


Figure 13


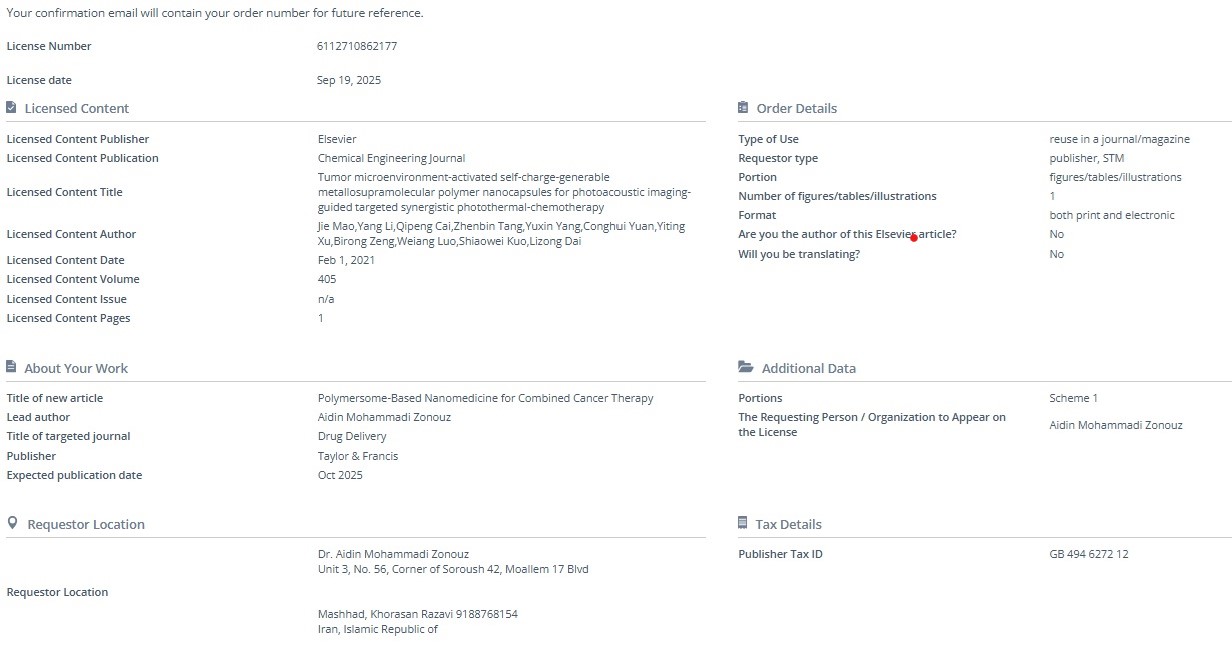

Supplement: Supplementary material — Permissions.docx [file IDRD_A_2577137_SM7569.docx]
